# Highly selective cesium(I) capture under acidic conditions by a layered sulfide

**DOI:** 10.1038/s41467-022-28217-8

**Published:** 2022-02-03

**Authors:** Jun-Hao Tang, Jian-Ce Jin, Wei-An Li, Xi Zeng, Wen Ma, Ji-Long Li, Tian-Tian Lv, Ying-Chen Peng, Mei-Ling Feng, Xiao-Ying Huang

**Affiliations:** 1 State Key Laboratory of Structural Chemistry, Fujian Institute of Research on the Structure of Matter, Chinese Academy of Sciences, Fuzhou, Fujian 350002 P. R. China; 2 University of Chinese Academy of Sciences, Beijing, 100049 P. R. China; 3 Fujian Province Joint Innovation Key Laboratory of Fuel and Materials in Clean Nuclear Energy System, Fujian Institute of Research on the Structure of Matter, Chinese Academy of Sciences, Fuzhou, Fujian 350002 P. R. China

**Keywords:** Materials chemistry, Nuclear chemistry, Pollution remediation, Solid-state chemistry

## Abstract

Radiocesium remediation is desirable for ecological protection, human health and sustainable development of nuclear energy. Effective capture of Cs^+^ from acidic solutions is still challenging, mainly due to the low stability of the adsorbing materials and the competitive adsorption of protons. Herein, the rapid and highly selective capture of Cs^+^ from strongly acidic solutions is achieved by a robust K^+^-directed layered metal sulfide KInSnS_4_ (InSnS-1) that exhibits excellent acid and radiation resistance. InSnS-1 possesses high adsorption capacity for Cs^+^ and can serve as the stationary phase in ion exchange columns to effectively remove Cs^+^ from neutral and acidic solutions. The adsorption of Cs^+^ and H_3_O^+^ is monitored by single-crystal structure analysis, and thus the underlying mechanism of selective Cs^+^ capture from acidic solutions is elucidated at the molecular level.

## Abstract (web summary)

The removal of radiocesium from acidic solutions is challenging. Here, the authors report the rapid and highly selective capture of cesium(I) from strongly acidic solutions by a robust layered metal sulfide.

## Introduction

With the rapid development of nuclear energy, growing concerns about its safety have been raised. ^137^Cs, as the fission product of ^235^U, is one of the main sources of radioactivity in spent fuel (1230 g/ton)^1^. ^137^Cs with a long half-life (*t*_1/2_ ~ 30.17 years) can emit *γ*-rays^1^. It is highly migratory in the environment, causing cell damage, cancer, and even death in humans^2^. Once radioactive cesium is released into the environment, it will be extremely polluting and cause harm to the entire ecosystem^3^. For example, the Fukushima nuclear accident released a large number of radioactive Cs^+^ ions into the environment, causing total radioactivity levels in some fish to remain above the limit (100 Bq/kg) until now^4^. Therefore, the disposal of ^137^Cs has received much attention due to its potential threat to the environment.

The strongly acidic high-level-liquid-wastes (HLLWs) resulting from the recovery of uranium and plutonium from spent fuel by the PUREX process contain ^137^Cs in ionic form^1,5–7^. The key for the disposal of HLLWs is the removal of strongly radioactive ions (such as ^137^Cs) to reduce their radioactivity level^6^. The separated and purified ^137^Cs can also be prepared as isotope sources and reused in agriculture and medical fields^3^. However, HLLWs are extremely complex, containing not only Cs^+^, but also Sr^2+^, Ln^3+^, Na^+^ and so on^6,8^. Such complex components would pose a huge challenge for the selective separation of Cs^+^ from HLLWs. Methods such as solvent extraction, chemical precipitation, and ion exchange/sorption have been used for the enrichment and separation of radiocesium^9^. These methods, however, have some disadvantages in terms of operation, cost, or selectivity, such as the expensive and toxic extractants used in solvent extraction and the great volume of radioactive sludge produced by precipitation^10^. Although ion exchange is considered an ideal method for controlling radioactive contamination due to its simplicity of operation, high efficiency, and lack of secondary contamination^11^, the development of stable and highly selective ion exchangers for the efficient capture of Cs^+^ in acidic solutions still remains a great challenge.

Due to the instability or poor selectivity of materials for Cs^+^ capture under acidic conditions, most of the current studies are restricted to that under neutral or weak acidic conditions^9,11,12^. In contrast, materials that can effectively remove Cs^+^ ions under extremely acidic condition are still very limited, exemplified mainly by ammonium phosphomolybdate and its complexes or cupric aromatic crown ether-modified silyl compounds^13–16^. In recent years, metal sulfides have been a very promising class of radioactive ion exchangers^12,17–20^ which display excellent removal performance for Cs^+^. However, the capture of Cs^+^ by metal sulfides under strongly acidic conditions (nitric acid concentrations >1 mol/L) is challenging, and in particular, the adsorption mechanism has been not clearly identified. Therefore, it is of vital significance to develop acid-tolerant ion exchangers that can selectively capture Cs^+^ from strongly acidic solutions for radioactive liquid waste treatment and to clarify the mechanism of Cs^+^ removal for revealing the structure–function relationship.

Herein, the strategy to improve stability of materials by introducing Sn^4+^ and In^3+^ with high valency and large radii into sulfides has been applied. Such an approach leads to a robust K^+^-directed layered metal sulfide KInSnS_4_ (denoted as InSnS-1) with excellent acid and irradiation resistances. Specifically, it can maintain its [InSnS_4_]_*n*_^*n*−^ layers even in 1–4 mol/L HNO_3_ solutions. InSnS-1 has high Cs^+^ ion-exchange capacity (*q*_m_^Cs^ = 316.0 mg/g in neutral solutions; *q*_m_^Cs^ = 98.6 mg/g in 1 mol/L HNO_3_ solutions). Under the coexistence of high-concentration competing ions such as Na^+^, Sr^2+^, and La^3+^, InSnS-1 exhibits high selectivity for Cs^+^ in acidic solutions, which endows InSnS-1 with excellent Cs-Sr or Cs-La separation in the acidic environment. Moreover, ion-exchange columns loaded with InSnS-1 can effectively treat neutral and acidic solutions containing high concentrations of Cs^+^ (190.13 and 84.25 mg/L, respectively) with treatment efficiencies up to 1300 and 650 bed volumes, respectively. The successful determination of the single-crystal structures of InSnS-1 and its three ion-exchange products help visualize the ion exchange between Cs^+^ or hydrated protons (H_3_O^+^) and interlayered K^+^ ions of InSnS-1. This is a systematic study of the selective Cs^+^ capture under strongly acidic condition with an emphasis on revealing the capture mechanism at the molecular level.

## Results

### Synthesis

Among the reported metal sulfide ion exchangers, K_2*x*_Mn_*x*_Sn_3−*x*_S_6_ (*x* = 0.5–0.95, KMS-1) and KInSn_2_S_6_ (KMS-5) have shown excellent acid resistance (retaining the parent structure at pH <1)^8,17,21^. A common feature of both compounds is the high coordination number of metal ions in the framework (i.e., metal ions are coordinated with six S atoms to form octahedral geometries), which is the highest among the reported metal sulfide ion exchangers^8,17,22^. The high coordination of metal ions may be beneficial to the structural stability of metal sulfides. In addition, it has been shown that the introduction of high-valency metal ions in the framework helps to resist the exchange of competing protons^8^. Therefore, we used the strategy of introducing high-valency, large-radius metal ions to synthesize metal sulfides with highly coordinated metal ions aiming to improve the acid stability of sulfide ion exchangers and their ability to selectively capture target ions. Thus, a robust K^+^-directed layered metal sulfide InSnS-1 has been synthesized by a simple solid-phase method according to the following reaction formula (Eq. (1)):

1 $${{{{\rm{KInS}}}}}_{2}+{{{\rm{Sn}}}}+2\,{{{\rm{S}}}}\,\mathop{\longrightarrow }\limits^{750{}^{\circ }{{{\rm{C}}}},4\,{{{\rm{days}}}}}\;{{{{\rm{KInSnS}}}}}_{4}$$

Although KInSnS_4_ with the same stoichiometric ratio has been reported, the previous synthesis methods yielded mixed phase of KInSnS_4_ with two distinct structures (*α*-KInSnS_4_ and *β*-KInSnS_4_)^23^. Moreover, the previous synthesis methods reported are operationally complex, requiring the use of CS_2_ vapor stream or K_2_CO_3_. Notably, when K_2_CO_3_ is used, the reaction mixture usually needs to be heated to a specific temperature to release carbon dioxide firstly before sealing the quartz tube. By contrast, KInS_2_ as a precursor was utilized here, which allows the synthesis of single-phase KInSnS_4_ (InSnS-1) on a large scale easily. Moreover, though the structure of InSnS-1 is similar with *α*-KInSnS_4_, InSnS-1 shows different K^+^ positions. The detail can be found in the following crystal structures part.

### Crystal structures and insight into the Cs^+^ removal mechanism under neutral and acidic conditions

The reported *α*-KInSnS_4_ and *β*-KInSnS_4_ both present two-dimensional (2D) anionic layer of [InSnS_4_]_*n*_^*n*−^ formed by [(In/Sn)S_6_] octahedra interconnected via edge sharing^23^. The difference is that in *α*-KInSnS_4_, the S atoms in the [(In/Sn)S_6_] octahedron are disordered with half-occupancy resulting in two alternative orientations for [InSnS_4_]_*n*_^*n*−^ layers (Supplementary Fig. 3)^23^. Accordingly, the interlayer disordered K^+^ ions are hexa-coordinated with S atoms to form KS_6_ with both triangular antiprismatic and prismatic orientations in *α*-KInSnS_4_, while only triangular antiprismatic KS_6_ are formed in *β*-KInSnS_4_^23^. Although it seems that InSnS-1 has similar structure as *α*-KInSnS_4_ (Supplementary Fig. 3), structural refinements of InSnS-1 show that the K^+^ ions reside in two different sites of K1 (1*b*) and K1B (2*d*), rather than only one site (1*b*) in *α*-KInSnS_4_ (Supplementary Fig. 4a, b).

The ion-exchange properties of *α*-KInSnS_4_ and *β*-KInSnS_4_, especially for the selective capture of Cs^+^ under strongly acidic condition have not been systematically investigated. In order to investigate the capture mechanism of Cs^+^ under neutral and acidic conditions, the single-crystal structures of ion-exchange products (InSnS-1-Cs, InSnS-1-Cs/H, InSnS-1-H) were analyzed and compared with that of the pristine InSnS-1. This allows us to visualize the mechanism of selective Cs^+^ capture by InSnS-1 and effects of protons on the process of Cs^+^ capture. Combining single-crystal structure analysis and EDS results, the ion-exchange reaction can be represented by the following equations (Eqs. (2)–(4)):

2 $${{{{{{\rm{KInSnS}}}}}}}_{4}+{{{{{{\rm{Cs}}}}}}}^{+}\to {{{{{{\rm{CsInSnS}}}}}}}_{4}({{{\mbox{InSnS\mbox{-}}}1\mbox{-}Cs}})+{{{{{{\rm{K}}}}}}}^{+}$$

3 $${{{{{{\rm{KInSnS}}}}}}}_{4}+\frac{1}{3}{{{{{{\rm{Cs}}}}}}}^{+}+\frac{2}{3}{{{{{{\rm{H}}}}}}}_{3}{{{{{{\rm{O}}}}}}}^{+}\to {{{{{{\rm{Cs}}}}}}}_{\frac{1}{3}}{({{{{{{\rm{H}}}}}}}_{3}{{{{{\rm{O}}}}}})}_{\frac{2}{3}}{{{{{{\rm{InSnS}}}}}}}_{4}({{{\mbox{InSnS\mbox{-}}}1\mbox{-}Cs}}/{{{{{\rm{H}}}}}})+{{{{{{\rm{K}}}}}}}^{+}$$

4 $${{{{{{\rm{KInSnS}}}}}}}_{4}+{{{{{{\rm{H}}}}}}}_{3}{{{{{{\rm{O}}}}}}}^{+}+{{{{{{\rm{H}}}}}}}_{2}{{{{{\rm{O}}}}}}\to ({{{{{{\rm{H}}}}}}}_{3}{{{{{\rm{O}}}}}}){{{{{{\rm{InSnS}}}}}}}_{4}\cdot {{{{{{\rm{H}}}}}}}_{2}{{{{{\rm{O}}}}}}({{{\mbox{InSnS\mbox{-}}}1\mbox{-}H}})+{{{{{{\rm{K}}}}}}}^{+}$$

**Fig. 1:**
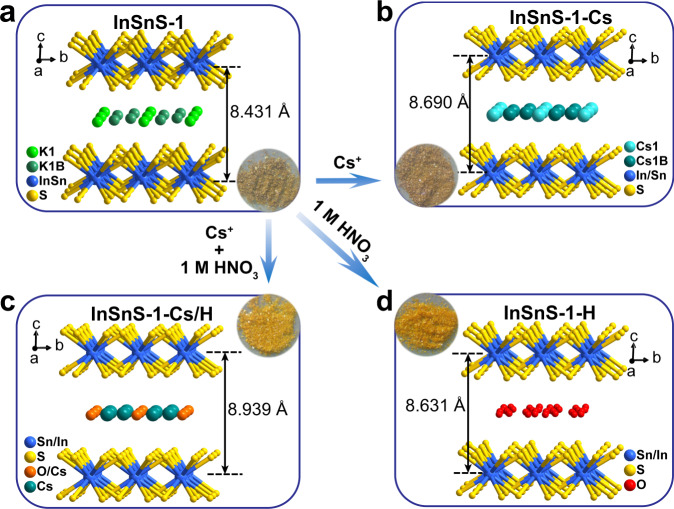
Crystal structures diagrams before and after ion exchange. View of the crystal structures of **a** InSnS-1, **b** InSnS-1-Cs, **c** InSnS-1-Cs/H, and **d** InSnS-1-H along *a* axis.

Detailed structural information of compounds is shown in Supplementary Tables 1–6. Compared with InSnS-1, both InSnS-1-Cs and InSnS-1-Cs/H exhibit an increase in the interlayer spacing (from 8.431 Å to 8.690 Å and 8.939 Å, respectively; Fig. 1a–c), which is attributed to the substitution of K^+^ (138 pm) by Cs^+^ with larger radius (170 pm)^24^. Although the radius of H_3_O^+^ (112 pm)^24^ is smaller than that of K^+^, the interlayer spacing of ${{\mbox{InSnS-}}}1{{\mbox{-H}}}$ still increases to 8.631 Å because of the entry of one lattice water molecule per formula. These results indicate that the interlayer spacing of InSnS-1 is tunable upon ion exchange and its layer structure shows flexibility even under acidic conditions, which contributes to the effective capture of Cs^+^.

It is noteworthy that each Cs^+^ ion is coordinated by six S atoms to form CsS_6_ with triangular antiprismatic and prismatic geometries in both InSnS-1-Cs and InSnS-1-Cs/H with Cs–S distances of 3.606 Å and 3.675 Å, respectively (Supplementary Fig. 5), which indicate strong interactions between S^2−^ and Cs^+^. Based on Pearson’s Hard-Soft-Acid-Base (HSAB) theory^25^, K^+^ is a hard acid whereas Cs^+^ is relatively softer. Thus, the soft basic S^2−^ from [InSnS_4_]_*n*_^*n*−^ layers has a stronger affinity for relatively soft acidic Cs^+^ than K^+^. Therefore, the flexible framework and the adjustable layer spacing of InSnS-1 as well as the strong interaction of Cs^+^ with S^2−^ play important roles for the rapid and highly selective capture of Cs^+^.

In addition, K^+^ ions can be completely exchanged by H_3_O^+^ ions under acidic conditions without Cs^+^ ions. H_3_O^+^ ions or water molecules become more disordered between the layers in InSnS-1-H (Fig. 1d). Although there have been studies on the capture of Cs^+^, the clear structural revelation is still scarce^26–29^. Especially, the microstructural illumination for the selective Cs^+^ capture under acidic conditions has not been reported. The current deep insight into the structural analyses facilitates the understanding of the mechanism of selective Cs^+^ capture by ion exchange and provides a reference for the design and synthesis of novel ion exchangers with the high selectivity for target radionuclides.

To further investigate the relationship between structures and properties of materials, a number of K^+^-directed layered or three-dimensional (3D) microporous sulfides including 3D-K_4_Cu_8_Ge_3_S_12_^30^, 2D-KCu_2_SbS_3_^31^, 2D-KYS_2_^32^. have been synthesized. It is a pity that our preliminary batch absorption experiments show that they have no Cs^+^ ion-exchange properties. To comparatively analyze the structure–function relationship, the reported structures including 2D-KMS-1^17,22^, 2D-KMS-2^18^, 2D-KTS-3^19^. and their ion-exchange properties have been also summarized. The shortest distances from K^+^ to anionic frameworks (noted as *D*_min_^K-S^) and the coordination numbers of K^+^ have been summarized (Supplementary Table 7).

In the case of 2D layered sulfides, 2D-KYS_2_ features an anionic layer of [YS_2_]_*n*_^*n*−^ built up by edge-sharing [YS_6_] octahedra^32^, which is similar to KMS ion exchangers^8,17,18^. However, 2D-KYS_2_ does not have ion-exchange properties for Rb^+^, Cs^+^, and Sr^2+^, even though 2D-RbYS_2_^32^ with the same layer structure as KYS_2_ has been reported. It is found that *D*_min_^K-S^ in 2D-KYS_2_ (3.174 Å) is much shorter than those in 2D-KMS-1 (3.353 Å), 2D-KMS-2 (3.494 Å), and 2D-KMS-5 (3.439 Å). 2D-*β*-K_2_CdSn_2_S_6_^33^ with short *D*_min_^K-S^ (3.157 Å) also cannot undergo ion-exchange reactions. Thus, the strong attraction between S^2−^ of anionic layers and K^+^ makes it difficult for K^+^ to escape. In the case of 3D microporous sulfides, 3D-K_3_Ga_3_Ge_7_S_20_ has a large *D*_min_^K-S^ (3.281 Å) but cannot accommodate other ions such as Cs^+^ due to small pore size excluding K^+^ (3.7 Å × 19.4 Å)^34^. Conversely, 3D-K_6_Sn[Zn_4_Sn_4_S_17_]^35^ has three different cavities, namely K1, K2, K3 cavities. The K^+^ ions (K3: 4 coordination, *D*_min_^K-S^ = 3.253 Å) in K3 cavity (diameter: ~4.1 Å) can be easily exchanged by Cs^+^ with strong selectivity. The K^+^ ions in the smaller K1 and K2 cavities (~3.0 Å; K1: 8 coordination, *D*_min_^K-S^ = 3.446 Å; K2: 6 coordination, *D*_min_^K-S^ = 3.144 Å), however, cannot be exchanged by Cs^+^ due to the limitation of the pore size.

Based on these facts, it can be inferred that *D*_min_^K-S^ is one of the main key factors in determining whether ion exchange occurs for layered sulfides. The smaller the value of *D*_min_^K-S^ is, the more attractive the anionic sulfide framework is for K^+^, and the less likely ion exchange will occur. Based on the currently available data, we can tentatively assume that ion exchange can be realized when *D*_min_^K-S^ is longer than 3.30 Å, and otherwise ion-exchange reaction difficulty occurs for layered sulfides. *D*_min_^K-S^ of 2D-InSnS-1 is 3.503 Å here, and thus the current compound exhibits excellent ion-exchange property. However, for 3D microporous compounds, the pore size and the coordination number of K^+^ are also necessary factors in addition to the *D*_min_^K-S^. If necessary, elevated reaction conditions will favor the promotion of ion-exchange reactions^35^. Of course, the universal conclusion remains to be analyzed in more depth with more examples.

### Characterizations of ion-exchange products

Single-crystal X-ray diffraction directly confirms that InSnS-1-Cs, InSnS-1-Cs/H, and InSnS-1-H can be obtained by ion exchange of InSnS-1 under different conditions. The successful ion exchange of K^+^ by Cs^+^ or H_3_O^+^ can be further corroborated by EDS, XPS, ICP. The X-ray powder diffraction patterns of InSnS-1 and its ion-exchange products were in high agreement with the corresponding single-crystal simulated ones (Supplementary Fig. 7). The elemental distribution mapping and EDS analysis results of ion-exchange products show that Cs^+^ ions are evenly distributed in the samples (Supplementary Figs. 8 and 9). As seen in the XPS spectra (Supplementary Fig. 10), two peaks of K 2*p* at 292.1 eV (K 2*p*_3/2_) and 294.8 eV (K 2*p*_1/2_) disappeared after Cs^+^ or H_3_O^+^ ion exchange, which confirms that K^+^ ions were completely exchanged. Conversely, the appearance of Cs 3*d*_5/2_ and Cs 3*d*_3/2_ peaks at 723.3 eV and 737.2 eV was observed in the XPS spectra of InSnS-1-Cs and InSnS-1-Cs/H^36^.

Compared to InSnS-1, InSnS-1-Cs has a significantly darker yellow color, while InSnS-1-Cs/H and InSnS-1-H display lighter yellow colors (Fig. 1), which correlates with the redshift and blueshift of their optical absorption edges, respectively. Compared to InSnS-1 (2.211 eV), the optical absorption edge of InSnS-1-Cs is slightly red-shifted to 2.147 eV, while InSnS-1-Cs/H and InSnS-1-H are blue-shifted to 2.255 and 2.220 eV, respectively (Supplementary Fig. 11). TG analysis (Supplementary Fig. 12) shows that InSnS-1 has good thermal stability. In addition, it can be seen that the exchanged products have obvious delamination compared with the pristine from the SEM images (Supplementary Figs. 13 and 14).

### Acid stability studies

**Fig. 2:**
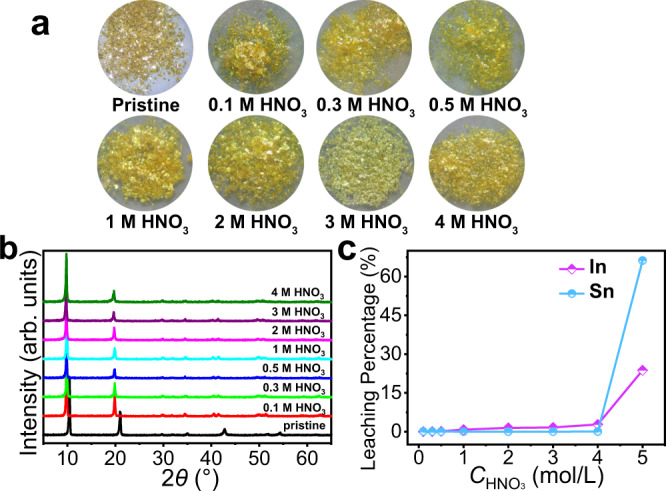
Acid stability studies of InSnS-1. **a** Photographs of pristine InSnS-1 and its crystals after immersion in different concentrations of nitric acid solutions. **b** X-ray powder diffraction spectra of InSnS-1 and its corresponding samples after soaking in different concentrations of nitric acid solutions. **c** In/Sn leaching percentage curves of InSnS-1. Detailed experimental data can be found in Supplementary Table 8.

A scavenger for Cs^+^ should be acid-stable as HLLWs are typically highly acidic^6^. The InSnS-1 crystals retained the crystallinity and the layered network after being immersed in highly concentrated (0.1–4 mol/L) acidic solutions for 10 h (Fig. 2a, b). The leaching rates of In^3+^ and Sn^4+^ in solution were less than 2.79% and 0.06%, respectively, which further confirms the high acid resistance of InSnS-1 (Fig. 2c). As the acidity increased further to 5 mol/L HNO_3_, the substance began to break down.

### Kinetic studies of Cs^+^ ion removal

**Fig. 3:**
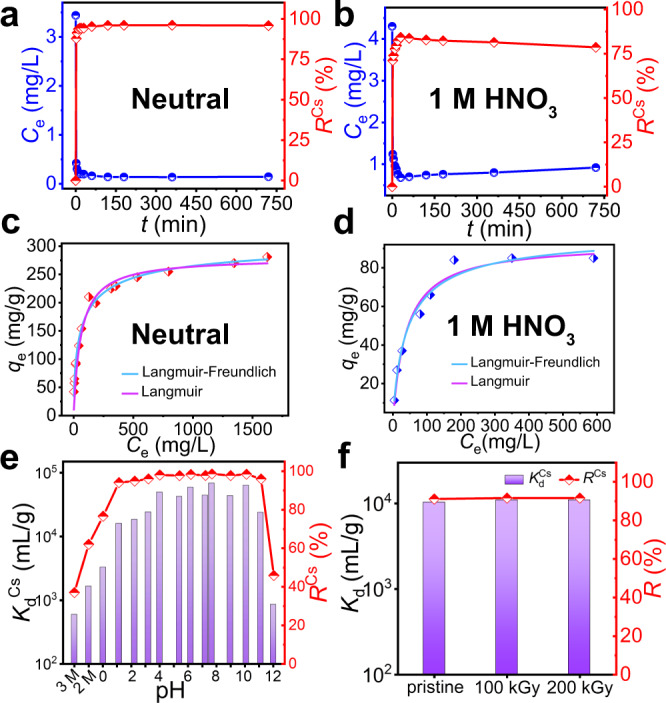
Adsorption kinetics, adsorption isotherms, pH-dependent studies, and irradiation stability studies of InSnS-1. Kinetics of Cs^+^ removal by InSnS-1 under **a** neutral and **b** 1 mol/L HNO_3_ solutions plotted as Cs^+^ concentration and the removal rate of Cs^+^ *vs* the time *t* (min), respectively. Equilibrium data for Cs^+^ ion exchange by InSnS-1 in **c** neutral and **d** 1 mol/L HNO_3_ solutions. **e** *K*_d_^Cs^ and *R*^Cs^ values of InSnS-1 at various initial pH values (2 M = 2 mol/L HNO_3_, 3 M = 3 mol/L HNO_3_, purple bar: *K*_d_^Cs^, red dotted line: *R*^Cs^). **f** *K*_d_^Cs^ and *R*^Cs^ values of the InSnS-1 samples before and after irradiation. Detailed experimental data can be found in Supplementary Tables 9–14.

It is essential that the ion exchanger is able to capture the target ions quickly. Therefore, kinetic experiments for the Cs^+^ capture by InSnS-1 in neutral and strongly acidic solutions were carried out. In neutral solution, the removal rate *R* (%) of Cs^+^ by InSnS-1 rapidly reached over 90% within 5 min (Fig. 3a). In 1 mol/L HNO_3_ solution, *R*^Cs^ also rapidly reached over 80% within 20 min (Fig. 3b). Such a fast and efficient removal of Cs^+^ under acidic conditions is rarely reported because ion-exchange competition with protons is a severe issue^8,20^. Compared to the pseudo-first-order kinetics model^37^, the kinetic data of Cs^+^ capture by InSnS-1 in neutral and 1 mol/L HNO_3_ solutions were better fitted with the pseudo-second-order kinetics model^37^ with high correlation coefficients *R*^2^ (>0.9999) (Supplementary Fig. 15), which proves that the adsorption process is chemical sorption^38^.

### Cs^+^ ion adsorption isotherm studies

The exchange capacities of InSnS-1 for Cs^+^ in neutral and 1 mol/L HNO_3_ solutions at room temperature (RT) have been measured by static batch experiments. The ion-exchange capacity can be determined by fitting the equilibrium data with the Langmuir and the Langmuir–Freundlich isotherm models^39^. The isotherm curves (Fig. 3c, d) were better fitted with the Langmuir–Freundlich isotherm model, with the correlation coefficients (*R*^2^) of 0.99 and 0.98 in neutral and 1 mol/L HNO_3_ solutions, respectively. The maximum exchange capacity (*q*_m_^Cs^) of InSnS-1 for Cs^+^ reached 316 mg/g in neutral solutions, which is higher than that of many common Cs^+^ adsorbents^9^, including bentonite (Turkish samples: 300.35 mg/g)^40^, zeolite (Turkish samples: 89.18 mg/g)^40^, ferricyanide (Prussian blue granules: 241 mg/g)^41^, carbon-based materials (GO: 76.9 mg/g)^42^, metal sulfides (KMS-1^17^: 226 mg/g, KTS-3^19^: 280 mg/g) and is also higher than that of the commercially available AMP-PAN^43^ (81 mg/g). It is noteworthy that InSnS-1 can still hold an exchange capacity for Cs^+^ of 98.6 mg/g in strongly acidic solutions (1 mol/L HNO_3_), which is even higher than that of KMS-1 at pH = 0.8 (~90 mg/g)^17^. Noteworthy is that the adsorption capacity of sulfide materials for Cs^+^ under such strongly acidic condition has not been found before.

### pH-dependent experiments

In general, the pH value of solution has an effect on the adsorption/ion exchange^20,44,45^. The majority of reported materials are unstable under strongly acidic conditions or their ion-exchange properties are significantly reduced due to the effects of protonation^8,20^. Some previously reported good-performance Cs^+^ ion-exchange metal sulfides have shown a significant decrease in their ion-exchange performance when the concentration of HNO_3_ just approaches 1 mol/L^18,19,46^. For example, KMS-2^18^, KTS-3^19^, and FJSM-SnS^46^ have reduced *K*_d_^Cs^ of around 10^3^ mL/g at pH = 3, 2, and 0.7, respectively. Therefore, pH-dependent experiments for the Cs^+^ removal by InSnS-1 were carried out. The distribution coefficient (*K*_d_) was calculated to describe the affinity of the material for the target ions.

InSnS-1 maintained excellent removal performance for Cs^+^ over a wide range of pH values from 1.14 to 11.14, with *R*^Cs^ >94% and *K*_d_^Cs^ values all above 10^4^ mL/g (Fig. 3e). The *K*_d_^Cs^ value was still 3.3 × 10^3^ mL/g (*R*^Cs^ = 76.6%) at pH = 0.02 and it reached 1.6 × 10^3^ mL/g (*R*^Cs^ = 62.1%) even in 2 mol/L HNO_3_ solution. These indicate that in strongly acidic solutions InSnS-1 still has the ability to remove Cs^+^. It is very rare that such high efficiency can be retained under such strongly acidic solutions. Impressive, InSnS-1 was able to maintain the framework stability during ion exchange over a wide pH range (pH = 0.02–12.02) and in strongly acidic solutions (2 and 3 mol/L HNO_3_), which was confirmed by PXRD (Supplementary Fig. 16). Conversely, excellent sulfide-based ion-exchange materials such as KMS-2^18^, KTS-3^19^, FJSM-GAS-1^47^, FJSM-GAS-2^47^, InS-1^48^, InS-2^49^, and K@GaSnS-1^50^ have the disrupted frameworks at pH = 2–3. Although KMS-1^22^ and FJSM-SnS^46^ are more acid-resistant, their frameworks could only remain stable at pH of around 0.7. When the acidity is further enhanced, KMS-1 retains its lamellar structure but the composition of the layer structure changes^21^. In a word, metal sulfides with high stability and excellent Cs^+^ ion-exchange properties under strongly acidic conditions are rare, while the excellent performance of InSnS-1 certainly highlights the feasibility of metal sulfides for Cs^+^ removal in acidic waste streams.

### Irradiation stability studies

Irradiation stability is one of the necessary properties for radioactive ion scavengers. Therefore, the Cs^+^ ion-exchange capacities of InSnS-1 samples after 100 kGy and 200 kGy *γ*-irradiation were investigated. The results demonstrate that InSnS-1 has excellent irradiation stability, maintaining stable structure and ion-exchange properties (*R*^Cs^ >91%, *K*_d_^Cs^ >10^4^ mL/g) after intense irradiation (Fig. 3f, Supplementary Fig. 17).

### Competitive experiments in the presence of interfering ions

**Fig. 4:**
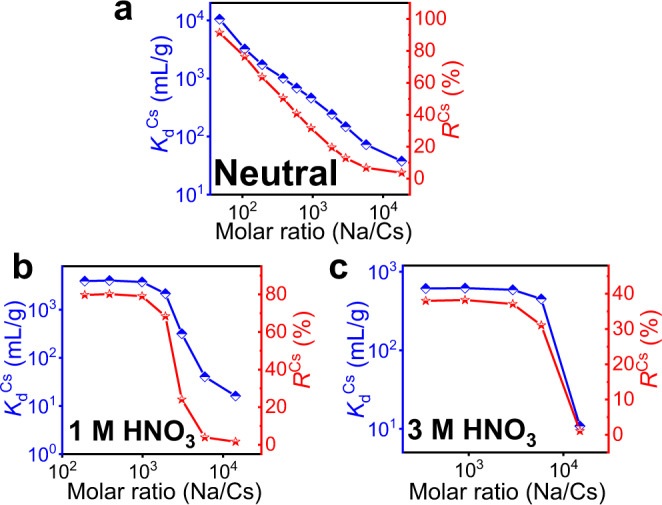
Selective capture of Cs^+^ by InSnS-1 in the coexistence of Na^+^ and Cs^+^. *K*_d_^Cs^ and *R*^Cs^ values of InSnS-1 in **a** neutral, **b** 1 mol/L HNO_3_, and **c** 3 mol/L HNO_3_ solutions with different Na/Cs molar ratios. Detailed experimental data can be found in Supplementary Table 15.

The selective extraction of Cs^+^ from HLLWs containing high concentrations of Na^+^ has been a major challenge because Na^+^ and Cs^+^ have similar hydration radii (219 pm for Na^+^ and 218 pm for Cs^+^)^8,24^. Therefore, the performances of InSnS-1 capturing Cs^+^ in neutral and acidic solutions with different Na/Cs molar ratios were investigated in detail. In neutral solutions, *K*_d_^Cs^ could still amount to 10^3^ mL/g when the Na/Cs molar ratio was 380 (*R*^Cs^ = 50.37%, Fig. 4a). In 1 mol/L HNO_3_ solutions, *K*_d_^Cs^ could still reach 2.16 × 10^3^ mL/g (*R*^Cs^ = 68.35%) even when the Na/Cs molar ratio was 1.92 × 10^3^ (Fig. 4b). Importantly, *R*^Cs^ and *K*_d_^Cs^ of InSnS-1 in 1 mol/L HNO_3_ solutions were higher than those in neutral solutions when the Na/Cs molar ratios were in the range from 190 to 3.07 × 10^3^, and the highest values of *K*_d_^Cs^ and *R*^Cs^ were 4.0 × 10^3^ mL/g and 79.71%, respectively (Supplementary Fig. 18). Even in 3 mol/L HNO_3_ solutions with Na/Cs molar ratios of about 2.9 × 10^3^ and 5.8 × 10^3^, *R*^Cs^ could still reach 37.11% and 31.06%, respectively, which were higher than *R*^Cs^ values in neutral or 1 mol/L HNO_3_ solutions under the same conditions (Fig. 4c, Supplementary Fig. 18). The separation factor *SF*_Cs/Na_ of InSnS-1 in 1 mol/L HNO_3_ solutions exceeded 100 when the Na/Cs molar ratios were 191, 387, and 983 (*SF*_Cs/Na_ were 172.3, 109.6, and 166.2, respectively). *SF* is related to the selectivity of the material which is used to measure whether the two ions can be well separated^18^. Excellent selectivity can be considered when the *SF* value is higher than 100^18^. Such excellent selectivity of InSnS-1 for Cs^+^ is attributed to that Na^+^ is hard acid, while Cs^+^ is softer acid compared to Na^+^. According to HSAB theory^25^, the soft base S^2−^ has a stronger affinity for Cs^+^ than Na^+^.

**Fig. 5:**
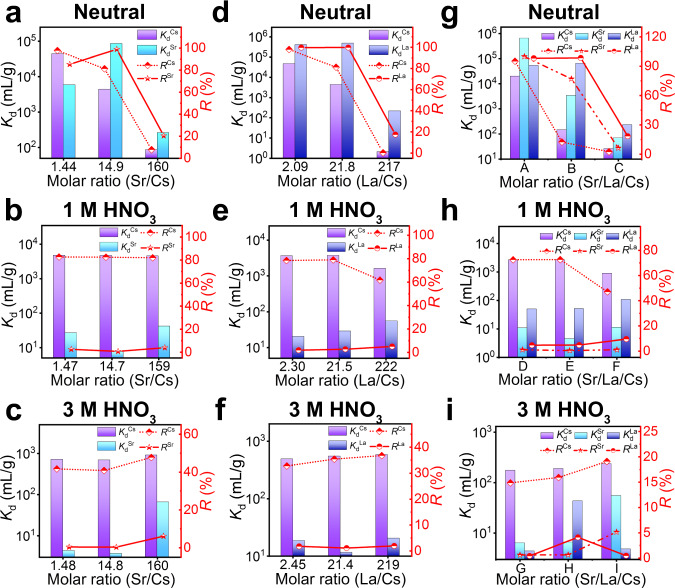
Selective capture of Cs^+^ by InSnS-1 in the presence of Sr^2+^ and/or La^3+^. *K*_d_ and *R* of Cs^+^ and Sr^2+^ ions removed by InSnS-1 in **a** neutral, **b** 1 mol/L HNO_3_, and **c** 3 mol/L HNO_3_ solutions with different Sr/Cs molar ratios. *K*_d_ and *R* of Cs^+^ and La^3+^ ions removed by InSnS-1 in **d** neutral, **e** 1 mol/L HNO_3_, and **f** 3 mol/L HNO_3_ solutions with different La/Cs molar ratios. *K*_d_ and *R* of Cs^+^, Sr^2+^, and La^3+^ ions removed by InSnS-1 in **g** neutral, **h** 1 mol/L HNO_3_, and **i** 3 mol/L HNO_3_ solutions with different Sr/La/Cs molar ratios (A = 1.38:1.27:1, B = 13.9:8.58:1, C = 123:73.9:1, D = 1.47:0.925:1, E = 13.6:8.45:1, F = 122:73.1:1, G = 1.45:0.880:1, H = 13.5:8.36:1, I = 123:73.7:1). Detailed experimental data can be found in Supplementary Tables 16–18.

The effect of some other ions in HLLWs (represented by Sr^2+^ and La^3+^) on the selective capture of Cs^+^ cannot be neglected; thus, extra experiments were performed^8^. In the individual competing ion system, the influence of solution acidity and competing ion concentrations on the performance of InSnS-1 in removing Cs^+^ is similar. Under neutral conditions, when the concentrations of Cs^+^ and competing Sr^2+^ or La^3+^ were low (i.e., the Sr/Cs molar ratios were 1.44 and 14.9 or the La/Cs molar ratios were 2.09 and 21.8), InSnS-1 possessed high *K*_d_ and *R* (above 10^3^ mL/g and 80%) for Cs^+^, Sr^2+^, La^3+^ ions, respectively (Fig. 5a, d). The maximum values of *K*_d_^Cs^, *K*_d_^Sr^, *K*_d_^La^ were 4.74 × 10^4^ mL/g, 8.57 × 10^4^ mL/g, 4.89 × 10^5^ mL/g, respectively, and the maximum removal rates of these ions were 97.93%, 98.85%, 99.80%, respectively. In 1 mol/L HNO_3_ solution, *K*_d_^Cs^ are still above 10^3^ mL/g with *R*^Cs^ around 80% even under very high concentrations of competing ions. By contrast, the removal performances of InSnS-1 for Sr^2+^ or La^3+^ in 1 mol/L HNO_3_ were significantly lower, with *K*_d_^M^ of 7.49–54.1 mL/g and *R*^M^ of 0.74%–5.13% (M = Sr or La) (Fig. 5b, e). Even in 3 mol/L HNO_3_ solutions containing high concentrations of competing Sr^2+^ or La^3+^, InSnS-1 still held *R*^Cs^ of 32.78%–47.74%, while *R*^M^ (M = Sr or La) were only in the range of 0.42%–6.15% (Fig. 5c, f).

Obviously, the enhancement of acidity has resulted in a greater attenuation in the removal efficiencies of Sr^2+^ and La^3+^ rather than Cs^+^. In 1 mol/L HNO_3_ solutions, *SF*_Cs/Sr_ (183.45, 630.87, and 110.70) exceeded 100 at three different Sr/Cs molar ratios, while *SF*_Cs/La_ also reached 184.31 and 133.93 when La/Cs molar ratios were 2.30 and 21.5, respectively. Even under 3 mol/L HNO_3_ condition, *SF*_Cs/Sr_ remained 170.00 and 189.97 at the Sr/Cs molar ratios of 1.48 and 14.8, respectively. The above results demonstrate that InSnS-1 can effectively achieve Cs-Sr (or Cs-La) separation in acidic solutions even though the concentration of Sr^2+^ (or La^3+^) is much higher than that of Cs^+^. Sulfide ion exchanger AgSnSe-1 can achieve Cs-Sr separation (*SF*_Cs/Sr_ = 121.4) in neutral solutions with low concentration of Cs^+^ and Sr^2+^ (both around 6 mg/L)^51^. However, to the best of our knowledge, no example of *SF*_Cs/Sr_ (or *SF*_Cs/La_) value that exceeds 100 in strongly acidic solution has been reported, where the Sr^2+^ (or La^3+^) ion concentration is much higher than the Cs^+^ ion concentration. Clearly, the current InSnS-1 is the first case for the excellent Cs-Sr or Cs-La separation in an acidic environment, which demonstrates again its excellent selectivity for Cs^+^ capture.

We also investigated the selective capture ability of InSnS-1 under the coexistence of Sr^2+^, La^3+^, and Cs^+^. In neutral solutions, their *K*_d_ (6.5 × 10^5^ mL/g, 5.3 × 10^4^ mL/g, 1.96 × 10^4^ mL/g, respectively) and *R* values (99.85%, 98.15%, 95.14%, respectively) were all very high when the Sr/La/Cs molar ratio was 1.38:1.27:1 (Fig. 5g). Nevertheless, in 1 mol/L HNO_3_ solution, *K*_d_^Cs^ could still reach 2.66 × 10^3^ mL/g with *R*^Cs^ of 72.71% when the Sr/La/Cs molar ratio was 13.6:8.45:1, whereas *K*_d_^Sr^ and *K*_d_^La^ were only 4.37 mL/g and 50.7 mL/g with low *R*^Sr^ and *R*^La^ of 0.43% and 4.83%, respectively (Fig. 5h). And when the Sr/La/Cs molar ratios were 1.47:0.925:1 and 13.6:8.45:1, *SF*_Cs/Sr_ retained 249.89 and 610.01, respectively. Even in the 3 mol/L HNO_3_ solution with high concentrations of La^3+^ and Sr^2+^ (the Sr/La/Cs molar ratio is 123:73.7:1), *R*^Cs^ could reach 19.1% which is significantly higher than *R*^Sr^ and *R*^La^ (5.17% and 0.47%) (Fig. 5i). The above results indicate that the selectivity of InSnS-1 for Cs^+^ is higher in acidic solution compared to in neutral solution.

Clearly, in neutral solutions with low ion concentrations InSnS-1 retains high removal efficiencies for all of Cs^+^, Sr^2+^, and La^3+^ in both individual competing ion system and mixed Sr^2+^, La^3+^, and Cs^+^ system (Fig. 5a, d, g). However, in acidic solutions, *K*_d_^Cs^ and *R*^Cs^ remain high and far exceed those of La^3+^ and Sr^2+^ ions even when the concentrations of La^3+^ and Sr^2+^ ions are much higher than that of Cs^+^. Besides, the increase in Na^+^, Sr^2+^ or La^3+^ concentration has less effect on Cs^+^ capture by InSnS-1 in strongly acidic solutions (1 and 3 mol/L HNO_3_ solutions) than in neutral solutions. Similarly, we obtained similar results in Cs^+^ selectivity experiments with different K/Cs or Ca/Cs molar ratios (Supplementary Fig. 19, Supplementary Tables 19 and 20) as well as in actual water sample experiments (Supplementary Note 3). Such results demonstrate that the presence of H_3_O^+^ is very favorable for enhancing the selective trapping ability of InSnS-1 for Cs^+^. This could also be attributed to the repulsive effect of H_3_O^+^ on Na^+^, Sr^2+^, and La^3+^. H_3_O^+^ has a small radius and is positively charged. Sr^2+^ and La^3+^ also have relatively small radii^24^ and highly positive charges compared to Cs^+^. Na^+^ has a smaller radius than Cs^+^, although it has the same positive charge as Cs^+^. Therefore, H_3_O^+^ has a relatively stronger repulsive effect on Na^+^, Sr^2+^, and La^3+^ which prevents them from entering the structure. Previous studies on the Cs^+^ ion exchangers with high acid resistance mainly focused on the exchange capacity of materials. By contrast, this work provides the first systematic study of the effect of divalent and trivalent ions on the selective capture of Cs^+^ under acidic conditions.

### Desorption and cycling experiments

To investigate how well the material can be recycled, desorption and recycling experiments were conducted. The 0.2 mol/L KCl solution or 1 mol/L HNO_3_ solution can effectively desorb Cs^+^ from InSnS-1-Cs (Supplementary Figs. 21 and 22). Due to the large adsorption capacity of InSnS-1 for Cs^+^ in 1 mol/L HNO_3_ solution, a small number of Cs^+^ were retained in the sample, but this problem could be solved by replacing the fresh HNO_3_ solution several times. However, to ensure the accuracy of the eluent concentration in the cyclic experiments, the HNO_3_ solution was not renewed during the desorption process. The experimental results show that the *R*^Cs^ can be maintained after three cycles, and the desorption rates are all about 100% and the layered structure of the material was maintained (Supplementary Figs. 23 and 24). The experimental results show that although part of Cs^+^ retaining in the structure affects the adsorption, the newly adsorbed Cs^+^ can be completely desorbed in each cycle. The results also indirectly suggest that the acidified material (i.e., the material after desorption in nitric acid solution) still possesses the removal capacity for Cs^+^.

### Column experiments

**Fig. 6:**
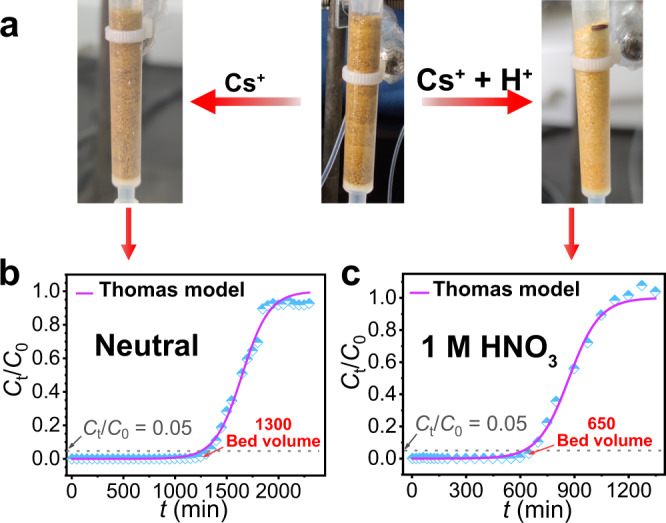
Dynamic capture of Cs^+^ by InSnS-1 as stationary phase in ion-exchange columns. **a** Photographs of the ion-exchange columns loaded with InSnS-1 before and after the column experiments under the neutral and acidic conditions. The Cs^+^ ion-exchange breakthrough curves of InSnS-1 under **b** neutral (*C*_Cs_ = 190.13 mg/L) and **c** 1 mol/L HNO_3_ conditions (*C*_Cs_ = 84.25 mg/L). Purple curves are fitted by the Thomas model. Detailed experimental data can be found in Supplementary Tables 26–28.

The breakthrough curves of Cs^+^ in the ion-exchange columns filled with InSnS-1 have been carried out under neutral and acidic conditions to evaluate the potential of InSnS-1 for industrial applications. The color of the adsorbent (InSnS-1 crystals) was darkened after treatment under neutral condition while it was lightened under acidic condition (Fig. 6a). The breakthrough curves were fitted to the Thomas model^52^ (correlation coefficient *R*^2^ >0.99) with a maximum adsorption capacity of 216.06 mg/g under neutral condition and 50.50 mg/g (Supplementary Table 28) under acidic condition (1 mol/L HNO_3_). Usually, a value of *C*_t_/*C*_0_ equal to 0.05 is used as a penetration point in industrial applications, taking into account the volume of wastewater treated^53^. For neutral and 1 mol/L HNO_3_ solutions with high concentrations of Cs^+^ (190.13 and 84.25 mg/L, respectively), the treatment volumes to reach the breakthrough points are approximately 1300 and 650 bed volumes, respectively (Fig. 6b, c).

InSnS-1 exhibits high dynamic adsorption capacity and large effective treatment volume for neutral solution containing high concentration of Cs^+^. Such excellent treatment capacity exceeds that of most reported Cs^+^ ion exchangers, e.g., MCC-g-AMP, M/SZMs, PEI/ZnFC^54–63^. Notably, hitherto studies on the column separation of Cs^+^ from strongly acidic solutions containing high concentration of Cs^+^ are still rare^16,64^. The current ion-exchange column provides a practical example that shows the effective removal of Cs^+^ from the highly acidic solution with high treatment amount. The above results demonstrate that InSnS-1 has great potential for practical application as a column packing in the field of radiocesium remediation.

## Discussion

In this work, the rapid and highly selective separation of Cs^+^ ions is achieved by the layered InSnS-1, especially for efficient capture of Cs^+^ even under highly acidic conditions. InSnS-1 as a Cs^+^ scavenger has the following main advantages: (i) excellent acid and irradiation resistances; (ii) fast adsorption kinetics and high adsorption capacities under neutral and acidic conditions; (iii) wide pH active range; (iv) effective separation of Cs^+^/M^*n*+^ (M^*n*+^ = Na^+^, Sr^2+^, and La^3+^) in acidic solutions; (v) easy desorption and reuse; (vi) effective dynamic separation of Cs^+^ as stationary phase in ion-exchange columns. The uptake mechanism of Cs^+^ and H_3_O^+^ is directly visualized through single-crystal structural analyses. All promising features of InSnS-1 for the Cs^+^ capture originate from the extremely strong interactions between soft S^2−^ of sulfide layers and relatively soft Cs^+^, the adjustable interlayer spacing, and structural flexibility of InSnS-1 even in acidic condition. In addition, our study elucidates the non-negligible facilitating role of H_3_O^+^ in the selective capture of Cs^+^ under acidic conditions. The important influence of the shortest K–S distance of K^+^-directed metal sulfides on the ion-exchange performance is also revealed. This work highlights metal sulfides for the selective capture of Cs^+^ ion from strongly acidic nuclear wastewaters and sheds light on the design of novel acid-tolerant sulfide-based ion-exchange materials for radiocesium decontamination with practical applications.

## Methods

### Starting materials

K_2_CO_3_ (AR, General-Reagent), S (CP, Kermel), Sn (99%, damas-beta), In (99.99%, CNBM (Cheng Du) Optoelectronic Materials Co., LTD), CsCl (99.99%, Shanghai Longjin Metal Materials Co., Ltd.), SrCl_2_·6H_2_O (AR, Tianjin Guangfu Reagent Co., Ltd.), LaCl_3_·7H_2_O (AR, Tianjin Guangfu Reagent Co., Ltd.), NaCl (AR, Sinopharm Chemical Reagent Co., Ltd), CaCl_2_·2H_2_O (74%, Shanghai Sili Chemical Plant), MgCl_2_ (AR, Adamas Reagent Co., Ltd). All the chemicals were used without further purification.

### Synthesis of InSnS-1

KInS_2_ precursor was firstly synthesized by hydrothermal method. A mixture of K_2_CO_3_ (9.0 mmol, 1.2439 g), In (9.0 mmol, 1.0334 g), S (30.0 mmol, 0.9618 g), and H_2_O (1.5 mL) was heated in a 28 mL polytetrafluoroethylene (PTFE) lined stainless steel autoclave at 220 °C for 2 days to obtain microcrystalline powder of KInS_2_. Then a mixture of KInS_2_ (3.0 mmol, 0.6541 g), Sn (3.0 mmol, 0.3561 g), S (6.0 mmol, 0.1924 g) was sealed under vacuum in a quartz tube. Then the quartz tube was heated to 750 °C in 4 h and maintained at 750 °C for 4 days, followed by program-controlled cooling to 550 °C for 3 days before turning off the furnace. After that, the quartz tube was naturally cooled to room temperature in the furnace. The crystals products were washed with deionized water and ethanol, and dried in natural condition. 1.2261 g of pure yellow flaky crystals of InSnS-1 could be obtained, which are stable in water and air. The higher actual yield than the theoretical value (1.2026 g) may be attributed to the water absorption of the compound.

### Syntheses of three ion-exchange crystal products

100 mg of InSnS-1 crystals were mixed with 100 mL neutral solution of 5000 mg/L Cs^+^ ions, 100 mL 1 mol/L nitric acid solution of 500 mg/L Cs^+^ ions, and 100 mL 1 mol/L HNO_3_ solution, respectively, which were shaken for 24 h at room temperature (RT). Then the crystals were washed with deionized water and ethanol, and dried naturally to afford the ion-exchange products formulated as CsInSnS_4_ (denoted as InSnS-1-Cs), Cs_1/3_(H_3_O)_2/3_InSnS_4_ (denoted as InSnS-1-Cs/H), and (H_3_O)InSnS_4_·H_2_O (denoted as InSnS-1-H), respectively.

### Characterizations

Single-crystal diffraction data for InSnS-1, InSnS-1-Cs, InSnS-1-Cs/H, and InSnS-1-H were collected with SuperNova CCD diffractometer with graphite monochromated Mo*Kα* (*λ* = 0.71073 Å). Powder X-ray diffraction (PXRD) patterns were obtained at RT by using a Miniflex II diffractometer with Cu*Kα* (*λ* = 1.54178 Å) at 30 kV and 15 mA in the angular range of 2*θ* = 5–65°. Energy dispersive spectroscopy (EDS), scanning electron microscope (SEM), and elemental distribution mapping analysis were carried out through a JEOL JSM-6700F scanning electron microscope. X-ray photoelectron spectroscopy (XPS) analysis was carried out through a ESCALAB 250Xi spectrometer with Al*K*α radiation. UV/visible spectra were obtained with a PerkinElmer Lambda 900 at RT. Thermo Gravimetric Analysis (TGA) was performed on a NETZSCH STA 449F3 DTA−TG analyzer. Ion concentrations in solutions were measured by inductively coupled plasma-mass spectroscopy (ICP-MS) or inductively coupled plasma-optical emission spectroscopy (ICP-OES). ICP-MS and ICP-OES tests were carried out by XSerise II and Thermo 7400, respectively. InSnS-1 crystal samples were irradiated with *γ*-rays at a total dose of 100 kGy (1.1 kGy/h for 95 h) and 200 kGy (1.1 kGy/h for 181.8 h) using a ^60^Co irradiation source (2 million curies) provided by Detection Center of Suzhou CNNC Huadong Radiation Co., Ltd, China.

### Acid stability experiments

In acid stability experiments, InSnS-1 crystals were immersed in different concentrations of HNO_3_ solutions for 10 h. After solid-liquid separation, the concentrations of leaching Sn and In in the solutions were determined by ICP-MS and the leaching percentages of Sn and In from the sample were inferred. PXRD of solid materials were measured to confirm whether the material framework remained stable. The equations and detailed descriptions used for data analysis can be found in Supplementary Note 2.

### Batch ion-exchange experiments

A typical ion-exchange experiment of InSnS-1 was carried out in a 20 mL glass bottle containing an aqueous solution (*V*/*m* = 1000 mL/g) of CsCl and InSnS-1 powder (obtained by grinding InSnS-1 crystals). The mixture was shaken in a shaker for 4 h at RT and then centrifuged for separation. The supernatant was filtered and diluted for ICP testing and the solid was washed several times with deionized water and anhydrous ethanol before drying. The equations and detailed descriptions used for data analysis can be found in Supplementary Note 2.

In isotherm experiments, different concentrations of Cs^+^ solutions under neutral or strongly acidic condition (1 mol/L HNO_3_) were prepared (44–1904 mg/L Cs^+^ at pH ~7; 4–590 mg/L Cs^+^ in 1 mol/L HNO_3_). In kinetics experiments, 50 mg of ground InSnS-1 crystal powder was added to 50 mL of 3.44 mg/L Cs^+^ solution or 50 mL of 1 mol/L HNO_3_ solution with 4.3 mg/L Cs^+^ under magnetic stirring at RT. The suspensions were sampled at different time intervals and then filtered to test the Cs^+^ concentrations in the solutions. In pH-dependent experiments, solutions with initial Cs^+^ concentrations of 4.6–4.88 mg/L at different acidity and alkalinity (pH = 0.02–12.04, 2 mol/L HNO_3_, 3 mol/L HNO_3_) were prepared. In the competitive ion-exchange experiments, Na^+^/Cs^+^, Sr^2+^/Cs^+^, La^3+^/Cs^+^, La^3+^/Sr^2+^/Cs^+^, Na^+^/Ca^2+^/Mg^2+^/Sr^2+^/Cs^+^ solutions at different acidities were prepared. The acidity or alkalinity of all solutions was adjusted by HNO_3_ and NaOH solutions. In actual water samples, Cs^+^ ions were added to river water (Fuzhou, Fujian and Longyan, Fujian) and seawater (Gulangyu, Xiamen, Fujian) to simulate Cs^+^ contaminated water bodies.

In the desorption experiments, the complete Cs^+^-exchanged samples (InSnS-1-Cs) were desorbed with 0.2 mol/L KCl solution or 1 mol/L HNO_3_ solution (*V*/*m* = 1000 mL/g, shaking for 12 h at RT). In the cycle experiments, 88.4 mg/L Cs^+^ solution was used for the adsorption and 1 mol/L HNO_3_ solution was used as the eluant. Overall, 3 cycles of adsorption-desorption were performed. The specific experimental procedures are shown in Supplementary Fig. 1 and detailed descriptions are given in Supplementary Note 1.

### Column experiments

The experimental setups are shown in Supplementary Fig. 2. 1.45 g of InSnS-1 crystal samples were packed into a 1 mL polyethylene column with an inner diameter of 0.56 cm and a packing height of ~4 cm. A sieve plate with the 50 μm pore size was put at the bottom of the column to avoid the loss of solid samples. Neutral solution with a concentration of 190.13 mg/L Cs^+^ and 1 mol/L HNO_3_ solution with 84.25 mg/L Cs^+^ passed through the ion-exchange column at a flow rate of 1 mL/min, respectively. The effluent solution sample within each 5 min was collected in a glass test tube and the measured concentration was approximated as the concentration at the intermediate moment. The solution flow rate and sample collection were controlled by the combined use of a peristaltic pump and an automatic collector.

## Supplementary information

Supplementary Information

## Data Availability

The single-crystal structures of InSnS-1, InSnS-1-Cs, InSnS-1-Cs/H, and InSnS-1-H are archived at the Cambridge Crystallographic Data Centre under the reference number CCDC-2107570, CCDC-2107571, CCDC-2107573, CCDC-2107572, respectively. These data can be obtained free of charge from The Cambridge Crystallographic Data Centre via www.ccdc.cam.ac.uk/data_request/cif. Any further relevant data are available from the authors upon reasonable request. The ion-exchange data generated in this study are provided in the Supplementary Information/Source Data file. Source data are provided with this paper.

## Source data

Source Data
