# Supplementary material for: Highly selective cesium(I) capture under acidic conditions by a layered sulfide
Source: Nat Commun. 2022 Feb 3;13:658. doi: 10.1038/s41467-022-28217-8 (PMC8813942; doi:10.1038/s41467-022-28217-8)
Supplement: Supplementary file 1 — Supplementary Information [file 41467_2022_28217_MOESM1_ESM.pdf]

Highly Selective Cesium(I) Capture under Acidic Conditions by a Layered Sulfide

Jun-Hao Tang,[1,2] Jian-Ce Jin,[1,2] Wei-An Li,[1,2] Xi Zeng,[1,2] Wen Ma,[1,2] Ji-Long Li,[1] Tian-Tian Lv,[1] Ying-Chen Peng,[1,2] Mei-Ling Feng*,[1,2,3] and Xiao-Ying Huang[1,2]

[1]State Key Laboratory of Structural Chemistry, Fujian Institute of Research on the Structure of Matter, Chinese Academy of Sciences, Fuzhou, Fujian 350002, P. R. China.

[2]University of Chinese Academy of Sciences, Beijing, 100049, P. R. China

[3]Fujian Province Joint Innovation Key Laboratory of Fuel and Materials in Clean Nuclear Energy System, Fujian Institute of Research on the Structure of Matter, Chinese Academy of Sciences, Fuzhou, Fujian 350002, P.R. China

*Corresponding author. Email: fml@fjirsm.ac.cn

Supplementary Note 1. Cycle experiment flow.

Cycle 0 was actually a sample preparation process for the following cycles; that is, the K^+ ions in InSnS-1 were fully exchanged by Cs^+ ions, and then the adsorbed Cs^+ ions were desorbed before the cycle experiment. 200 mg of InSnS-1 was firstly mixed with 200 mL of 5000 mg/L Cs^+ ion solution in a glass bottle and then the mixture was shaken for 12 h. Then the solid sample was washed with deionized water and anhydrous ethanol. The above solid sample was added into 200 mL of 1 mol/L HNO_3 solution and the mixture was shaken for 12 h (the HNO_3 solution would be renewed once at 6 h). After that, the solid sample (denoted as Sample A) was washed and dried before being used as the adsorbent for Cycle 1. *x* mg of Sample A and *x* mL of 88.4 mg/L Cs^+ ions solution were mixed and the mixture was shaken for 4 hours. The resulting solid sample (denoted as Sample B) was washed and dried. *y* mg of Sample B and *y* mL of 1 mol/L HNO_3 solutions were mixed which was shaken for 12 hours. After that, the resulting solid sample (denoted as Sample C) was washed and dried before being used as the adsorbent for Cycle 2. The concentrations of Cs^+ ions in solutions were measured after each adsorption or desorption.

Supplementary Note 2. Equation.

(1) Leaching rate (R_L):

$$R_{\mathrm{L}} = \frac{C_{\mathrm{P}}}{C_{\mathrm{T}}} \times 100\% \qquad (1)$$

where R_L(%) is the leaching rate, C_P (mg/L) is the concentration of In or Sn ions in the solution after soaking the samples, and C_T (ppm) is the theoretical concentration of In or Sn ions when the sample is completely dissolved in the solution.

(2) Removal rate (R):

$$R = \frac{(C_0 - C_{\mathrm{e}})}{C_{\mathrm{e}}} \times 100\% \qquad (2)$$

where C_0 (mg/L) and C_e (mg/L) are the initial and equilibrium concentration of target ions, respectively.

(3) Kinetics model[17]:

Pseudo-first-order kinetics model:

$$\ln (q_{\mathrm{e}} - q_{\mathrm{t}}) = \ln q_{\mathrm{e}} - k_1 t \qquad (3)$$

Pseudo-second-order kinetics model:

$$\frac{t}{q_t} = \frac{1}{k_2 q_{\mathrm{e}}^2} + \frac{t}{q_{\mathrm{e}}} \qquad (4)$$

where q_e (mg/g) and q_t (mg/g) are the ion exchange capacities at equilibrium and time *t* (min), respectively. k_1 (min^{-1}) and k_2 (g mg^{-1} min^{-1}) are pseudo-first-order and pseudo-second-order rate constants of kinetics models, respectively.

(4) Adsorption isotherm models[18]:

$$q_e = q_{\mathrm{m}} \frac{bC_{\mathrm{e}}}{1 + bC_{\mathrm{e}}} \qquad (5)$$

$$q_e = q_{\mathrm{m}} \frac{(bC_{\mathrm{e}})^{1/n}}{(1 + bC_{\mathrm{e}})^{1/n}} \qquad (6)$$

where C_e (mg/L) is the concentration at adsorption equilibrium, q_e (mg/g) is the amount of cation adsorbed per unit of adsorbent at adsorption equilibrium, q_m is the maximum adsorption capacity of the adsorbent, *b* (L/mg) is the Langmuir constant which is relevant to the free energy of exchange, and *n* is the Freundlich constant.

(5) Distribution coefficient (K_d):

$$K_{\mathrm{d}} = \frac{V}{m} \frac{(C_0 - C_{\mathrm{e}})}{C_{\mathrm{e}}} \qquad (7)$$

where C_0 (mg/L) and C_e (mg/L) are the initial and equilibrium concentrations of the target ion, respectively; *m* (g) and *V* (mL) are the mass of the ion exchange material and the volume of the solution used in the ion exchange experiment respectively.

(6) Separation factor (*SF*):

$$SF = \frac{K_{\mathrm{d}}^{\mathrm{A}}}{K_{\mathrm{d}}^{\mathrm{B}}} \qquad (8)$$

where K_d^A (mg/L) and K_d^B (mg/L) are the distribution coefficient of A and B ion, respectively.

(7) Desorption rate (*E*):

Considering adsorption-desorption as a complete cycle, the desorption rate in each cycle is closely related to the adsorption rate. Considering the loss of solids due to washing and drying during the cycle, the solids are weighed before each addition of the solution. The exchange of Cs^+ ions with H_3O^+ ions will cause a change in the weight of the material. Considering the above factors, the corresponding data need to be corrected when calculating the desorption rate (Here, the effects caused by the entry and exit of water molecules into and out of the structure are ignored.). The desorption rate can be considered as the percentage of the mass of Cs^+ ions desorbed off to the mass of Cs^+ ions adsorbed on, but considering the loss of solid mass, it can be calculated by the following equation:

$$E = \frac{C_{\mathrm{e}}^{\mathrm{de}} V_{\mathrm{de}}}{m_2 (m_{\mathrm{Cs}}^{\mathrm{ad}} / m_{\mathrm{ad}})} \times 100\% \qquad (9)$$

where C_e^{de} (mg/L) is the Cs^+ concentration in the solution after desorption, and V_{de} (L) is the volume of solution used during desorption. m_{Cs}^{ad} (mg) is the mass of adsorbed Cs^+ ion during the adsorption process, m_{ad} (mg) is the mass of the solid sample after adsorption of Cs^+, and m_2 (mg) is the mass of the solid sample used during desorption (Here, both solid samples refer to the sample obtained after the adsorption process, but m_2 is not equal to m_{ad} due to mass loss during washing and drying, so m_2 needs to be weighed to determine while m_{ad} needs to be calculated using the correction formula Supplementary Equation 11.). In Supplementary Equation 9, m_{Cs}^{ad}/m_{ad} represents the ratio of the mass of adsorbed Cs^+ ions to the total mass of adsorbent at the end of the adsorption process. And m_{Cs}^{ad} can be calculated using the following equation:

$$m_{\mathrm{Cs}}^{\mathrm{ad}} = \left(C_0^{\mathrm{ad}} - C_{\mathrm{e}}^{\mathrm{ad}}\right) V_{\mathrm{ad}} \qquad (10)$$

where C_0^{ad} (mg/L) and C_e^{ad} (mg/L) are the initial and adsorption equilibrium concentrations of Cs in solution during the adsorption process, respectively. V_{ad} (L) is the volume of the solution added during the adsorption. In addition, considering that the exchange of Cs^+ with H_3O^+ makes the total weight of the exchange product not equal to the mass of the added material, it is necessary to correct m_{ad} by the following equation:

$$m_{\mathrm{ad}} = m_1 + m_{\mathrm{Cs}}^{\mathrm{ad}} - \mathrm{M}_{\mathrm{H_3O^+}} \times m_{\mathrm{Cs}}^{\mathrm{ad}} / \mathrm{M}_{\mathrm{Cs^+}} \qquad (11)$$

where M_{Cs^+} = 132.9 and $M_{H_3O^+}$ = 19. Supplementary Equation 11 means that the mass of the solid at the end of the adsorption process (m_{ad}) is equal to the mass of the adsorbent added (m_1) plus the mass of the adsorbed Cs^+ ions (m_{Cs}^{ad}) and minus the mass of the hydrated protons exchanged out ($M_{H_3O^+} \times m_{Cs}^{ad}/M_{Cs^+}$).

(8) Thomas models[19-20]:

$$\frac{C_{\mathrm{t}}}{C_0} = \frac{1}{1 + \exp(K_{\mathrm{T}} q_{\mathrm{e}} m / Q - K_{\mathrm{T}} C_0 t)} \qquad (12)$$

where C_0 (mg/L) and C_t (mg/L) are the concentrations at the initial and time *t* (min), respectively. K_T (L min^{-1} mg^{-1}) is the rate constant in the Thomas model. q_e (mg/g) is the maximum adsorption capacity of the adsorbent, *Q* (L/min) is the volume flow rate, and *m* (g) is the mass of the adsorbent in the exchange column.

Supplementary Note 3. Discussion on Actual Water Sampling Experiments. Small amounts of Cs^+ ions were added to actual water samples to simulate contaminated water bodies, and the efficiency of InSnS-1 for the removal of Cs^+ in actual water environments had been investigated. The different initial concentrations of Cs^+ in the same water sample

had little effect on removal rates and K_d values due to the small amount of Cs^+ ions. K_d^{Cs} was higher than 2×10^4 mL/g and R^{Cs} was higher than 96% in river water sample 1, while K_d^{Cs} was higher than 4×10^3 mL/g and R^{Cs} was higher than 81% in river water sample 2. This was due to the lower concentrations of competing ions in river water sample 1 than in river water sample 2 (Supplementary Table 21). However, it was found that R^{Cs} and K_d^{Cs} of InSnS-1 in seawater are significantly lower compared with those in river water (Supplementary Figure 20a, Supplementary Table 21). This might be related to factors such as the complexity of seawater composition and further increases in competing ion concentrations. Therefore, the selective removal of Cs^+ by InSnS-1 was investigated in complex neutral or acidic solutions with the coexistence of Cs^+ and high concentrations of Ca^{2+}, Mg^{2+}, Na^+ ions which usually exist in actual water systems. Both K_d^{Cs} and R^{Cs} were low in neutral solution (1.0×10^2 mL/g and 9.45%, respectively), suggesting that the capture of Cs^+ ion by InSnS-1 is affected when the concentrations of interfering ions (Ca^{2+}, Mg^{2+}, and Na^+) are relatively high. However, as expected, the selectivity of InSnS-1 for Cs^+ increased significantly in 1 mol/L HNO_3 solution, with K_d^{Cs} of 2.84×10^3 mL/g and R^{Cs} of 73.98%, which were two to three orders of magnitude those of interfering ions (Supplementary figure 20b, Supplementary Table 22). Therefore, InSnS-1 maintains the outstanding selectivity for Cs^+ even in the complex and acidic environment with Ca^{2+}, Mg^{2+}, and Na^+ ions.

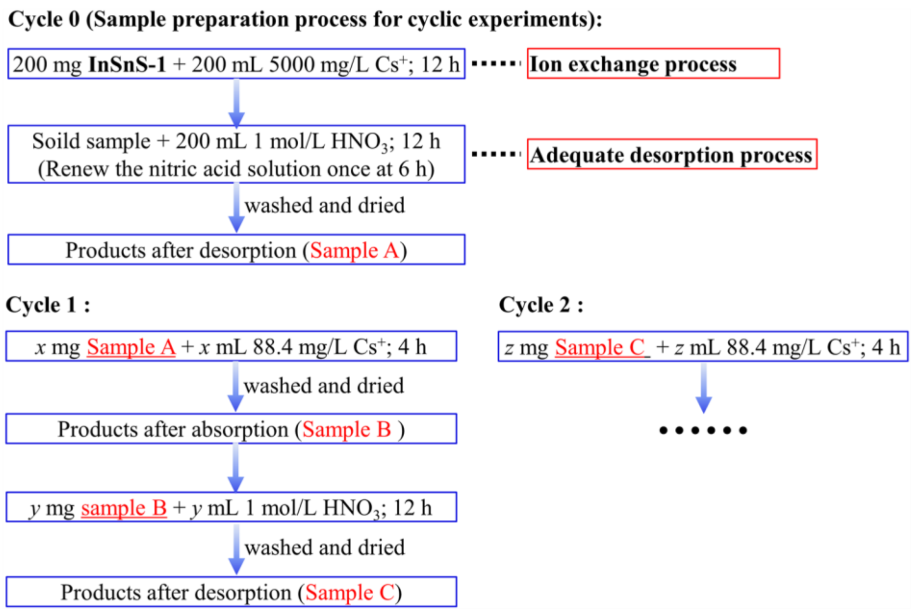

Supplementary Figure 1. Flow chart for the cycle experiments.

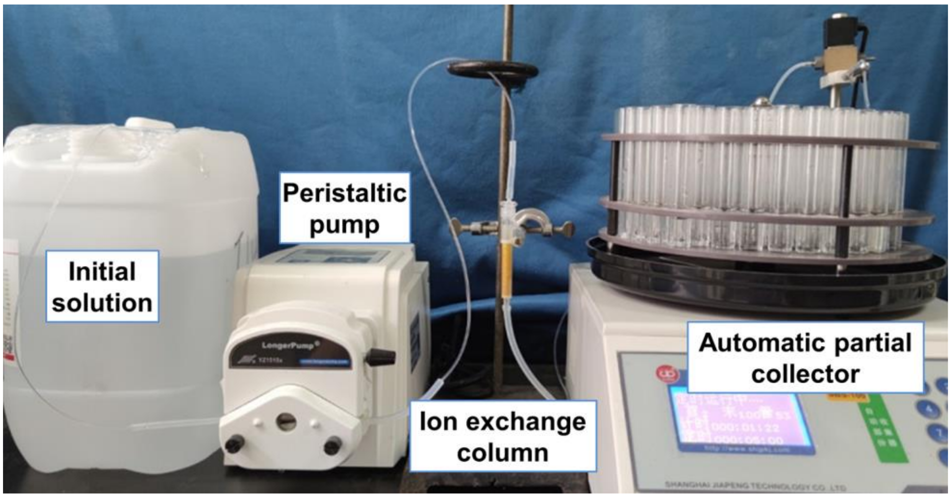

Supplementary Figure 2. Experimental setup for ion exchange column experiments (Loaded crystal sample dimensions: 0.1-0.9 mm).

Supplementary Table 1. Crystallographic data and structural refinements for compounds InSnS-1, InSnS-1-Cs, InSnS-1-Cs/H and InSnS-1-H.

Compound	InSnS-1	InSnS-1-Cs	InSnS-1-Cs/H	InSnS-1-H
Empirical formula	$K_{0.5}In_{0.5}Sn_{0.5}S_2$	$Cs_{0.5}In_{0.5}Sn_{0.5}S_2$	$Cs_{0.17}In_{0.5}Sn_{0.5}S_2HO_{0.33}$	$In_{0.5}Sn_{0.5}S_2H_{2.5}O$
Formula weight	200.43	247.33	209.76	199.39
Temperature/K	295(2)	100(2)	100(2)	293(2)
Wavelength/Å	0.71073	0.71073	0.71073	0.71073
Crystal system	hexagonal	hexagonal	hexagonal	hexagonal
Space group	$P6/m$	$P6/m$	$P6/m$	$P6/m$
a/Å	3.7159(7)	3.7170(12)	3.6620(10)	3.6941(12)
b/Å	3.7159(7)	3.7170(12)	3.6620(10)	3.6941(12)
c/Å	8.431(5)	8.690(4)	8.939(10)	8.631(10)
V/ Å^3	100.82(7)	103.98(8)	103.82(13)	102.00(14)
Z	1	1	1	1
$D_{calc.}$ (mg/m^3)	3.301	3.950	3.355	3.246
F (000)	91	109	94	92
Crystal size/mm^3	0.20 × 0.10 × 0.02	0.13 × 0.08 × 0.01	0.12 × 0.06 × 0.01	0.20 × 0.15 × 0.02
θ range for data collection/ °	2.416 to 29.707	2.344 to 29.775	2.278 to 29.431	2.360 to 29.608
Limiting indices	-4<=h<=4, -5<=k<=4, -10<=l<=11	-5<=h<=4, -4<=k<=5, -11<=l<=11	-5<=h<=3, -4<=k<=4, -11<=l<=12	-5<=h<=4, -5<=k<=4, -10<=l<=12
Completeness to θ = 25.242 °	100%	100%	100%	100%
Refinement method	Full-matrix least-squares on F^2	Full-matrix least-squares on F^2	Full-matrix least-squares on F^2	Full-matrix least-squares on F^2
Data / restraints / parameters	103 / 12 / 10	104 / 0 / 9	107 / 0 / 11	107 / 12 / 13
Goodness-of-fit on F^2	1.079	1.168	1.192	1.044
Final R indices [$I > 2\sigma(I)$]	R_1 = 0.0413, wR_2 = 0.1056	R_1 = 0.0439, wR_2 = 0.1044	R_1 = 0.0637, wR_2 = 0.1413	R_1 = 0.0457, wR_2 = 0.1012
R indices (all data)	R_1 = 0.0416, wR_2 = 0.1062	R_1 = 0.0476, wR_2 = 0.1073	R_1 = 0.0774, wR_2 = 0.1504	R_1 = 0.0558, wR_2 = 0.1139
CCDC	2107570	2107571	2107573	2107572

[a] $R_1 = \sum\|F_o| - |F_c\|/\sum|F_o|$, $wR_2 = [\sum w(F_o^2 - F_c^2)^2/\sum w(F_o^2)^2]^{1/2}$.

Supplementary Table 2. Selected bond lengths (Å) and angles (°) for compound InSnS-1.

K(1)-S(1)#1	3.503(5)	K(1)-S(1)#11	3.503(5)	M(1)-S(1)#15	2.587(3)
K(1)-S(1)#2	3.503(5)	K(1B)-S(1)#13	3.503(5)	M(1)-S(1)#8	2.587(3)
K(1)-S(1)#3	3.503(5)	K(1B)-S(1)#12	3.503(5)	M(1)-S(1)#16	2.587(3)
K(1)-S(1)#4	3.503(5)	K(1B)-S(1)#2	3.503(5)	M(1)-S(1)#4	2.587(3)
K(1)-S(1)#5	3.503(5)	K(1B)-S(1)#3	3.503(5)	M(1)-S(1)#17	2.587(3)
K(1)-S(1)#6	3.503(5)	K(1B)-S(1)#6	3.503(5)	M(1)-S(1)#2	2.587(3)
K(1)-S(1)#7	3.503(5)	K(1B)-S(1)#7	3.503(5)	M(1)-S(1)	2.587(3)
K(1)-S(1)#8	3.503(5)	K(1B)-K(1B)#8	3.7159(7)	M(1)-S(1)#18	2.587(3)
K(1)-S(1)	3.503(5)	M(1)-S(1)#14	2.587(3)	M(1)-S(1)#10	2.587(3)
K(1)-S(1)#9	3.503(5)	M(1)-S(1)#6	2.587(3)	M(1)-S(1)#19	2.587(3)
K(1)-S(1)#10	3.503(5)				
S(1)#14-M(1)-S(1)#6	180.0(2)	S(1)#16-M(1)-S(1)#2	180.0(2)	S(1)#6-M(1)-S(1)#10	91.81(14)
S(1)#15-M(1)-S(1)#8	180.0(2)	S(1)#15-M(1)-S(1)	88.19(14)	S(1)#16-M(1)-S(1)#10	88.19(14)
S(1)#14-M(1)-S(1)#16	91.81(14)	S(1)#8-M(1)-S(1)	91.81(14)	S(1)#2-M(1)-S(1)#10	91.81(14)
S(1)#6-M(1)-S(1)#16	88.19(14)	S(1)#4-M(1)-S(1)	91.81(14)	S(1)#14-M(1)-S(1)#19	91.81(14)
S(1)#15-M(1)-S(1)#4	88.19(14)	S(1)#17-M(1)-S(1)	88.19(14)	S(1)#6-M(1)-S(1)#19	88.19(14)
S(1)#8-M(1)-S(1)#4	91.81(14)	S(1)#15-M(1)-S(1)#18	91.81(14)	S(1)#16-M(1)-S(1)#19	91.81(14)
S(1)#15-M(1)-S(1)#17	91.81(14)	S(1)#8-M(1)-S(1)#18	88.19(14)	S(1)#2-M(1)-S(1)#19	88.19(14)
S(1)#8-M(1)-S(1)#17	88.19(14)	S(1)#4-M(1)-S(1)#18	88.19(14)	S(1)#10-M(1)-S(1)#19	180.0(2)
S(1)#4-M(1)-S(1)#17	180.0(2)	S(1)#17-M(1)-S(1)#18	91.81(14)	In(1)-S(1)-In(1)#20	91.81(14)
S(1)#14-M(1)-S(1)#2	88.19(14)	S(1)-M(1)-S(1)#18	180	In(1)-S(1)-In(1)#21	91.81(14)
S(1)#6-M(1)-S(1)#2	91.81(14)	S(1)#14-M(1)-S(1)#10	88.19(14)	In(1)#20-S(1)-In(1)#21	91.81(14)

(M = 0.5 In + 0.5 Sn)

Symmetry transformations used to generate equivalent atoms: #1 *x*+1, *y*, -*z*+1; #2 -*x*-1, -*y*, *z*; #3 -*x*, -*y*+1, -*z*+1; #4 *x*, *y*-1, *z*; #5 *x*, *y*-1, -*z*+1; #6 -*x*, -*y*+1, *z*; #7 -*x*-1, -*y*, -*z*+1; #8 *x*+1, *y*, *z*; #9 *x*, *y*, -*z*+1; #10 -*x*, -*y*, *z*; #11 -*x*, -*y*, -*z*+1; #12 -*x*-1, -*y*+1, -*z*+1; #13 -*x*-1,-*y*+1,*z*; #14 *x*, *y*-1, -*z*; #15 -*x*-1, -*y*, -*z*; #16 *x*+1, *y*, -*z*; #17 -*x*, -*y*+1, -*z*; #18 –*x* ,-*y*, -*z*; #19 *x*, *y*, -*z*; #20 *x*-1, *y*, *z*; #21 *x*, *y*+1, *z*.

Supplementary Table 3. Selected bond lengths (Å) and angles (°) for compound InSnS-1-Cs.

Cs(1)-S(1)#1	3.606(5)	Cs(1)-S(1)#11	3.606(5)	M(1)-S(1)#15	2.588(4)
Cs(1)-S(1)#2	3.606(5)	Cs(1B)-S(1)#13	3.606(5)	M(1)-S(1)#6	2.588(4)
Cs(1)-S(1)#3	3.606(5)	Cs(1B)-S(1)#12	3.606(5)	M(1)-S(1)#16	2.588(4)
Cs(1)-S(1)#4	3.606(5)	Cs(1B)-S(1)#2	3.606(5)	M(1)-S(1)#4	2.588(4)
Cs(1)-S(1)#5	3.606(5)	Cs(1B)-S(1)#3	3.606(5)	M(1)-S(1)#2	2.588(4)
Cs(1)-S(1)#6	3.606(5)	Cs(1B)-S(1)#6	3.606(5)	M(1)-S(1)#17	2.588(4)
Cs(1)-S(1)#7	3.606(5)	Cs(1B)-S(1)#7	3.606(5)	M(1)-S(1)	2.588(4)
Cs(1)-S(1)#8	3.606(5)	Cs(1B)-Cs(1B)#4	3.7170(12)	M(1)-S(1)#18	2.588(4)
Cs(1)-S(1)	3.606(5)	M(1)-S(1)#14	2.588(4)	M(1)-S(1)#10	2.588(4)
Cs(1)-S(1)#9	3.606(5)	M(1)-S(1)#8	2.588(4)	M(1)-S(1)#19	2.588(4)
Cs(1)-S(1)#10	3.606(5)				
S(1)#14-M(1)-S(1)#8	180	S(1)#4-M(1)-S(1)#17	180	S(1)#6-M(1)-S(1)#10	91.80(16)
S(1)#15-M(1)-S(1)#6	180	S(1)#14-M(1)-S(1)	88.20(16)	S(1)#16-M(1)-S(1)#10	88.20(16)
S(1)#15-M(1)-S(1)#16	91.80(16)	S(1)#8-M(1)-S(1)	91.80(16)	S(1)#2-M(1)-S(1)#10	91.80(16)
S(1)#6-M(1)-S(1)#16	88.20(16)	S(1)#4-M(1)-S(1)	91.80(16)	S(1)#15-M(1)-S(1)#19	91.80(16)
S(1)#14-M(1)-S(1)#4	88.20(16)	S(1)#17-M(1)-S(1)	88.20(16)	S(1)#6-M(1)-S(1)#19	88.20(16)
S(1)#8-M(1)-S(1)#4	91.80(16)	S(1)#14-M(1)-S(1)#18	91.80(16)	S(1)#16-M(1)-S(1)#19	91.80(16)
S(1)#15-M(1)-S(1)#2	88.20(16)	S(1)#8-M(1)-S(1)#18	88.20(16)	S(1)#2-M(1)-S(1)#19	88.20(16)
S(1)#6-M(1)-S(1)#2	91.80(16)	S(1)#4-M(1)-S(1)#18	88.20(16)	S(1)#10-M(1)-S(1)#19	180
S(1)#16-M(1)-S(1)#2	180	S(1)#17-M(1)-S(1)#18	91.80(16)	In(1)-S(1)-In(1)#20	91.80(16)
S(1)#14-M(1)-S(1)#17	91.80(16)	S(1)-M(1)-S(1)#18	180	In(1)-S(1)-In(1)#21	91.80(16)
S(1)#8-M(1)-S(1)#17	88.20(16)	S(1)#15-M(1)-S(1)#10	88.20(16)	In(1)#20-S(1)-In(1)#21	91.80(16)

(M = 0.5 In + 0.5 Sn)

Symmetry transformations used to generate equivalent atoms: #1 *x*+1, *y*, -*z*+1; #2 -*x*-1, -*y*, *z*; #3 -*x*, -*y*+1, -*z*+1; #4 *x*, *y*-1, *z*; #5 *x*, *y*-1, -*z*+1; #6 -*x*, -*y*+1, *z*; #7 -*x*-1, -*y*, -*z*+1; #8 *x*+1, *y*, *z*; #9 *x*, *y*, -*z*+1; #10 -*x*, -*y*, *z*; #11 -*x*, -*y*, -*z*+1; #12 -*x*-1, -*y*+1, -*z*+1; #13 -*x*-1, -*y*+1, *z*; #14 -*x*-1, -*y*, -*z*; #15 *x*, *y*-1, -*z*; #16 *x*+1, *y*, -*z*; #17 -*x*, -*y*+1, -*z*; #18 -*x*, -*y*, -*z*; #19 *x*, *y*, -*z*; #20 *x*-1, *y*, *z*; #21 *x*, *y*+1, *z*.

Supplementary Table 4. Selected bond lengths (Å) and angles (°) for compound InSnS-1-Cs/H.

M(1)-S(2)	2.571(6)	M(1)-S(2)#9	2.571(6)	Cs(1)-Cs(1)#19	3.6620(10)
M(1)-S(2)#1	2.571(6)	M(1)-S(2)#10	2.571(6)	Cs(1)-Cs(1)#14	3.6620(10)
M(1)-S(2)#2	2.571(6)	M(1)-S(2)#11	2.571(6)	Cs(1)-Cs(1)#4	3.6620(10)
M(1)-S(2)#3	2.571(6)	S(2)-Cs(2)#13	3.675(9)	Cs(1)-Cs(1)#20	3.6620(10)
M(1)-S(2)#4	2.571(6)	S(2)-Cs(1)#14	3.675(9)	Cs(1)-Cs(1)#6	3.6620(10)
M(1)-S(2)#5	2.571(6)	S(2)-Cs(1)	3.675(9)	Cs(2)-Cs(2)#4	3.6620(10)
M(1)-S(2)#6	2.571(6)	S(2)-Cs(2)#15	3.675(9)	Cs(2)-Cs(2)#6	3.6620(10)
M(1)-S(2)#7	2.571(6)	Cs(1)-Cs(1)#18	3.6620(10)	Cs(2)-Cs(2)#19	3.6620(10)
M(1)-S(2)#8	2.571(6)				

S(2)#1-M(1)-S(2)#2	180.0(4)	S(2)#6-M(1)-S(2)#7	180	S(2)#8-M(1)-S(2)#10	90.8(3)
S(2)-M(1)-S(2)#3	89.2(3)	S(2)#1-M(1)-S(2)#8	89.2(3)	S(2)#1-M(1)-S(2)#11	90.8(3)
S(2)-M(1)-S(2)#4	90.8(3)	S(2)#2-M(1)-S(2)#8	90.8(3)	S(2)#2-M(1)-S(2)#11	89.2(3)
S(2)#3-M(1)-S(2)#4	180.0(4)	S(2)#5-M(1)-S(2)#8	180.0(4)	S(2)#5-M(1)-S(2)#11	90.8(3)
S(2)#1-M(1)-S(2)#5	90.8(3)	S(2)-M(1)-S(2)#9	180	S(2)#8-M(1)-S(2)#11	89.2(3)
S(2)#2-M(1)-S(2)#5	89.2(3)	S(2)#3-M(1)-S(2)#9	90.8(3)	S(2)#10-M(1)-S(2)#11	180.0(4)
S(2)-M(1)-S(2)#6	90.8(3)	S(2)#4-M(1)-S(2)#9	89.2(3)	Sn(1)#20-S(2)-Sn(1)	90.8(3)
S(2)#3-M(1)-S(2)#6	89.2(3)	S(2)#6-M(1)-S(2)#9	89.2(3)	Sn(1)#20-S(2)-In(1)	90.8(3)
S(2)#4-M(1)-S(2)#6	90.8(3)	S(2)#7-M(1)-S(2)#9	90.8(3)	Sn(1)#20-S(2)-Sn(1)#14	90.8(3)
S(2)-M(1)-S(2)#7	89.2(3)	S(2)#1-M(1)-S(2)#10	89.2(3)	Sn(1)-S(2)-Sn(1)#14	90.8(3)
S(2)#3-M(1)-S(2)#7	90.8(3)	S(2)#2-M(1)-S(2)#10	90.8(3)	In(1)-S(2)-Sn(1)#14	90.8(3)
S(2)#4-M(1)-S(2)#7	89.2(3)	S(2)#5-M(1)-S(2)#10	89.2(3)		

(M = 0.5 In + 0.5 Sn)

Symmetry transformations used to generate equivalent atoms: #1 *x*, *y*-1, -*z*; #2 -*x*, -*y*+1, *z*; #3 -*x*-1, -*y*, -*z*; #4 *x*+1, *y*, *z*; #5 *x*+1, *y*, -*z*; #6 *x*, *y*-1, *z*; #7 -*x*, -*y*+1, -*z*; #8 -*x*-1, -*y*, *z*; #9 -*x*, -*y*, -*z*; #10 -*x*, -*y*, *z*; #11 *x*, *y*, -*z*; #12 -*x*-1, -*y*+1, *z*; #13 -*x*-1, -*y*+1, -*z*+1; #14 *x*, *y*+1, *z*; #15 -*x*, -*y*+1, -*z*+1; #16 -*x*-1, -*y*, -*z*+1; #17 -*x*, -*y*, -*z*+1; #18 *x*+1, *y*+1, *z*; #19 *x*-1, *y*-1, *z*; #20 *x*-1, *y*, *z*.

Supplementary Table 5. Selected bond lengths (Å) and angles (°) for compound InSnS-1-H.

M(1)-S(1)#1	2.579(4)	M(1)-S(1)#5	2.579(4)	M(1)-S(1)#8	2.579(4)
M(1)-S(1)#2	2.579(4)	M(1)-S(1)#6	2.579(4)	M(1)-S(1)#9	2.579(4)
M(1)-S(1)#3	2.579(4)	M(1)-S(1)#7	2.579(4)	M(1)-S(1)#10	2.579(4)
M(1)-S(1)#4	2.579(4)	M(1)-S(1)	2.579(4)	M(1)-S(1)#11	2.579(4)
S(1)#1-M(1)-S(1)#2	180	S(1)#2-M(1)-S(1)#8	91.50(17)	S(1)#2-M(1)-S(1)#11	88.50(17)
S(1)#3-M(1)-S(1)#4	180	S(1)#5-M(1)-S(1)#8	88.50(17)	S(1)#5-M(1)-S(1)#11	91.50(17)
S(1)#1-M(1)-S(1)#5	91.50(17)	S(1)#6-M(1)-S(1)#8	91.50(17)	S(1)#6-M(1)-S(1)#11	88.50(17)
S(1)#2-M(1)-S(1)#5	88.50(17)	S(1)#3-M(1)-S(1)#9	91.50(17)	S(1)#8-M(1)-S(1)#11	180.0(2)
S(1)#1-M(1)-S(1)#6	88.50(17)	S(1)#4-M(1)-S(1)#9	88.50(17)	Sn(1)#13-S(1)-Sn(1)#14	91.50(17)
S(1)#2-M(1)-S(1)#6	91.50(17)	S(1)#7-M(1)-S(1)#9	88.50(17)	Sn(1)#13-S(1)-Sn(1)	91.50(17)
S(1)#5-M(1)-S(1)#6	180	S(1)-M(1)-S(1)#9	91.50(17)	Sn(1)#14-S(1)-Sn(1)	91.50(17)
S(1)#3-M(1)-S(1)#7	88.50(17)	S(1)#3-M(1)-S(1)#10	88.50(17)	H(1)-O(1)-H(2)	109.7
S(1)#4-M(1)-S(1)#7	91.50(17)	S(1)#4-M(1)-S(1)#10	91.50(17)	H(1)-O(1)-H(3)	108.1
S(1)#3-M(1)-S(1)	91.50(17)	S(1)#7-M(1)-S(1)#10	91.50(17)	H(2)-O(1)-H(3)	108.9
S(1)#4-M(1)-S(1)	88.50(17)	S(1)-M(1)-S(1)#10	88.50(17)	H(2A)-O(2)-H(2B)	109.3
S(1)#7-M(1)-S(1)	180.0(2)	S(1)#9-M(1)-S(1)#10	180.0(2)	H(2A)-O(2)-H(2C)	106.8
S(1)#1-M(1)-S(1)#8	88.50(17)	S(1)#1-M(1)-S(1)#11	91.50(17)	H(2B)-O(2)-H(2C)	106.8

(M = 0.5 In + 0.5 Sn.)

Symmetry transformations used to generate equivalent atoms: #1 *x*, *y*, -*z*+2; #2 -*x*, -*y*, *z*; #3 *x*-1, *y*-1, *z*; #4 -*x*+1, -*y*+1, -*z*+2; #5 *x*-1, *y*-1, -*z*+2; #6 -*x*+1, -*y*+1, *z*; #7 -*x*, -*y*, -*z*+2; #8 -*x*+1, -*y*, *z*; #9 *x*-1, *y*, *z*; #10 -*x*+1, -*y*, -*z*+2; #11 *x*-1, *y*, -*z*+2; #12 -*x*+2, -*y*+1, *z*; #13 *x*+1, *y*, *z*; #14 *x*+1, *y*+1, *z*.

Supplementary Table 6. Atomic coordinates (× 10^4), equivalent isotropic displacement parameters (Å^2 × 10^3), SOFs and atomic sites for InSnS-1, InSnS-1-Cs, InSnS-1-Cs/H and InSnS-1-H. U(eq) is defined as one third of the trace of the orthogonalized U^{ij} tensor.

Compound	Atom	*x*	*y*	*z*	*U*(eq)	SOF	site
	In(1)	0	0	0	16(1)	0.5	1a
	Sn(1)	0	0	0	16(1)	0.5	1a
**InSnS-1**	S(1)	-3333	3333	1715(6)	14(1)	0.5	4h
	K(1)	0	0	5000	91(13)	0.24(3)	1b
	K(1B)	-3333	3333	5000	91(13)	0.130(14)	2d
	In(1)	0	0	0	6(1)	0.5	1a
	Sn(1)	0	0	0	6(1)	0.5	1a
**InSnS-1-Cs**	S(1)	-3333	3333	1665(7)	7(1)	0.5	4h
	Cs(1)	0	0	5000	66(4)	0.245(7)	1b
	Cs(1B)	-3333	3333	5000	66(4)	0.127(4)	2d
	Sn(1)	0	0	0	24(1)	0.5	1a
	In(1)	0	0	0	24(1)	0.5	1a
	S(2)	-3333	3333	1637(11)	26(2)	0.5	4h
**InSnS-1-Cs/H**	Cs(1)	0	0	5000	90(60)	0.012(19)	1b
	O(1)	0	0	5000	90(60)	0.3334	1b
	Cs(2)	-3333	3333	5000	89(19)	0.077(10)	2d
	Sn(1)	0	0	1	31(1)	0.5	1a
	In(1)	0	0	1	31(1)	0.5	1a
**InSnS-1-H**	S(1)	6667	3333	8321(7)	27(1)	0.5	4h
	O(1)	1400(700)	2400(500)	5000	90(40)	0.12(6)	6k
	O(2)	6667	3333	5000	90(40)	0.14(6)	2d

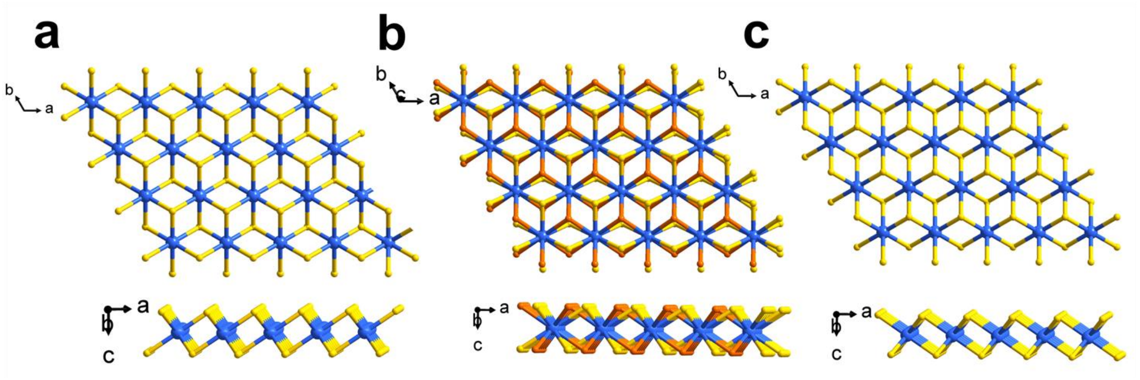

Supplementary Figure 3. Diagrams of the single-layer structure of $[InSnS_4]_n^{n-}$ in α-$KInSnS_4$ and InSnS-1 with (a) and without (b) removal of one orientation of layer and in β-$KInSnS_4$ (c). Blue balls: In/Sn atoms, yellow or orange balls: S atoms.

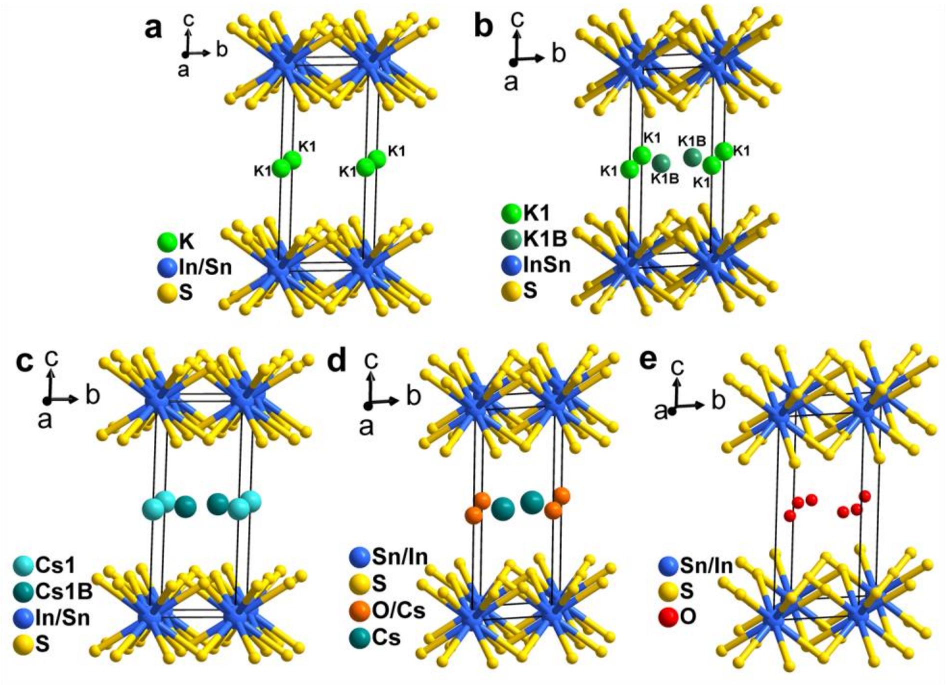

Supplementary Figure 4. Structural diagrams of (a) α-$KInSnS_4$, (b) InSnS-1, (c) InSnS-1-Cs, (d) InSnS-1-Cs/H and (e) InSnS-1-H showing the occupation positions for interlayer cations. Cs^+ ions enter the structure under neutral conditions resulting in InSnS-1-Cs in which Cs^+ ions are disordered in the interlayer and occupy the original K1 (1*b*) and K1B (2*d*) positions in InSnS-1. However, H_3O^+ ions besides Cs^+ ions are also found in the interlayer of InSnS-1-Cs/H under acidic conditions (1 mol/L HNO_3). And H_3O^+ or water molecules tend to occupy the original K1 (1*b*) positions, while Cs^+ ions prefer to occupy two positions of original K1 (1*b*) and K1B (2*d*).

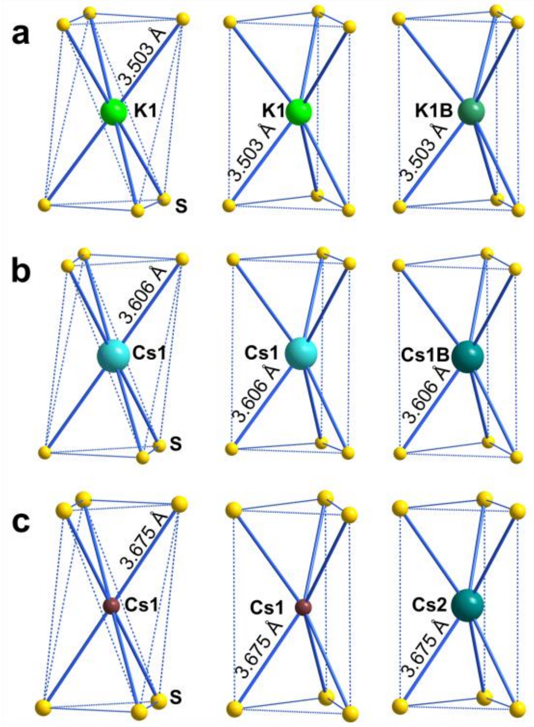

Supplementary Figure 5. Coordination modes of K^+ or Cs^+ ion in (a) InSnS-1, (b) InSnS-1-Cs and (c) InSnS-1-Cs/H. For clarity, only S atoms coordinated to the corresponding K^+ or Cs^+ are shown.

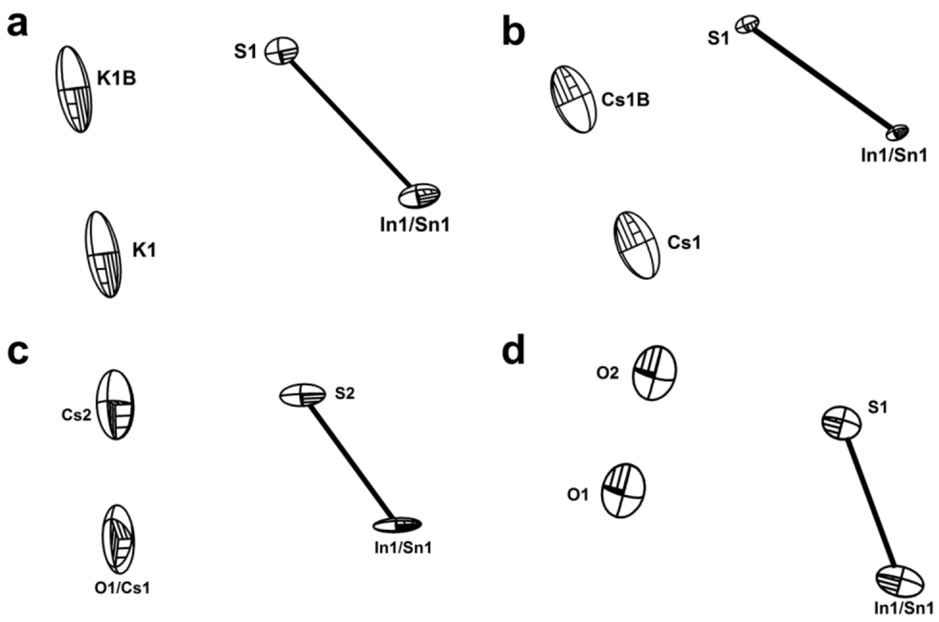

Supplementary Figure 6. *ORTEP* drawings (50% ellipsoid probability) of the asymmetric units of (a) InSnS-1 and (b) InSnS-1-Cs. *ORTEP* drawings (50% ellipsoid probability) of the non-hydrogen atoms in asymmetric units of (c) InSnS-1-Cs/H and (d) InSnS-1-H.

Supplementary Table 7. Minimum K-S distances and ion exchange properties of reported K^+-directed sulfide materials.

Compound	D*	K-S category	Shortest distance (Å)	Coordination number (K with S)	Exchangeable ions	Ref.
$KInSnS_4$ (**InSnS-1**)	2	K1-S	3.503	6	Cs^+	This work
		K1B-S	3.503	6		
$K_{2x}Mn_xSn_{3-x}S_6$ (x = 0.5-0.95, KMS-1)	2	K1-S	3.514	6	Cs^+, Sr^{2+}, Rb^+, UO_2^{2+}	1-3
		K2-S	3.353	4		
$K_{2x}Mg_xSn_{3-x}S_6$ (x = 0.5−1, KMS-2)	2	K1-S	3.494	6	Cs^+, Sr^{2+}, Ni^{2+}, Ag^+, Hg^{2+}	4-5
		K2-S	3.494	6		
$KInSn_2S_6$ (KMS-5)	2	K1-S	3.439	4	^{241}Am, ^{152}Eu	6
		K2-S	3.586	6		
$K_{2x}Sn_{4-x}S_{8-x}$ (x = 0.65–1, KTS-3)	2	K1-S	3.387	6	Cs^+, Sr^{2+}, UO_2^{2+} etc.	7
		K2-S	3.425	6		
$K_6Sn[Zn_4Sn_4S_{17}]$	3	K1-S	3.446	8	Cs^+, Rb^+, NH_4^+	8
		K2-S	3.144	6		
		K3-S	3.253	4		
$K_x[Bi_{4-x}Mn_xS_6]$	2	K1-S	3.456	6	Cs^+, Sr^{2+}, Rb^+ etc.	9
		K2-S	3.456	6		
$K_4Cu_8Ge_3S_{12}$	3	K1-S	3.223	4	no ion exchange properties found yet	10
$K_2CdSn_2S_6$ (β)	2	K1-S	3.197	7		11
		K2-S	3.154	7		
$K_2Ag_3Sb_3S_7$	2	K1-S	3.217	9		12
KCu_2SbS_3	2	K1-S	3.173	7		13
		K2-S	3.195	8		
KYS_2	2	K1-S	3.174	6		14
$K_2Ag_6Sn_3S_{10}$	3	K1-S	3.082	6		15
$K_3Ga_3Ge_7S_{20}$	3	K1-S	3.281	7		16
		K2-S	3.338	6		

* D = Structural dimensionality of the anionic sulfide network.

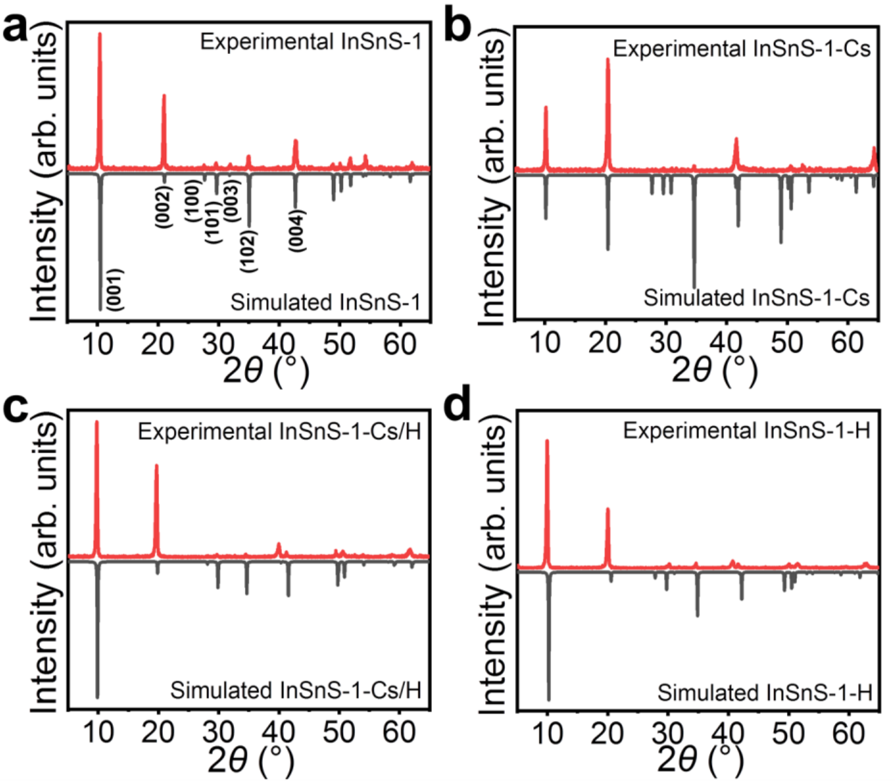

Supplementary Figure 7. Experimental and simulated PXRD patterns of (a) InSnS-1, (b) InSnS-1-Cs, (c) InSnS-1-Cs/H and (d) InSnS-1-H.

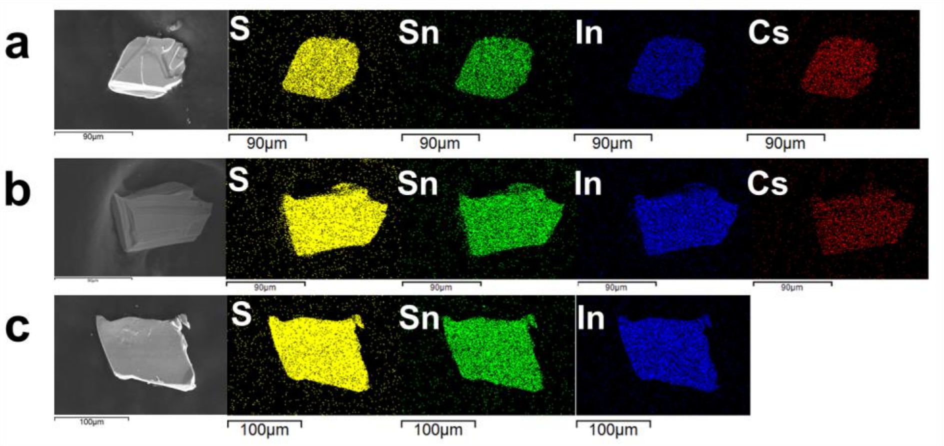

Supplementary Figure 8. Elemental distribution maps of (a) InSnS-1-Cs, (b) InSnS-1-Cs/H and (c) InSnS-1-H.

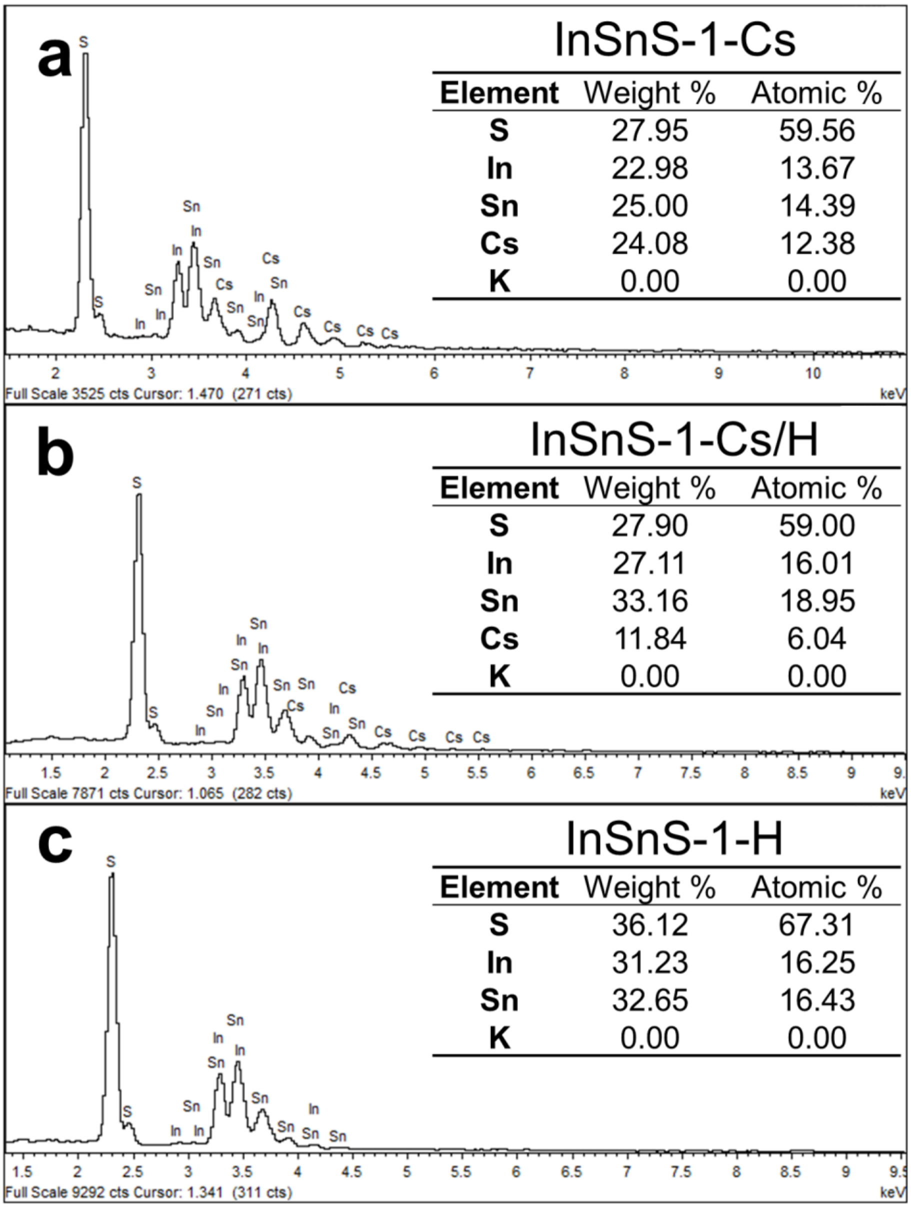

Supplementary Figure 9. EDS analysis results of (a) InSnS-1-Cs, (b) InSnS-1-Cs/H and (c) InSnS-1-H.

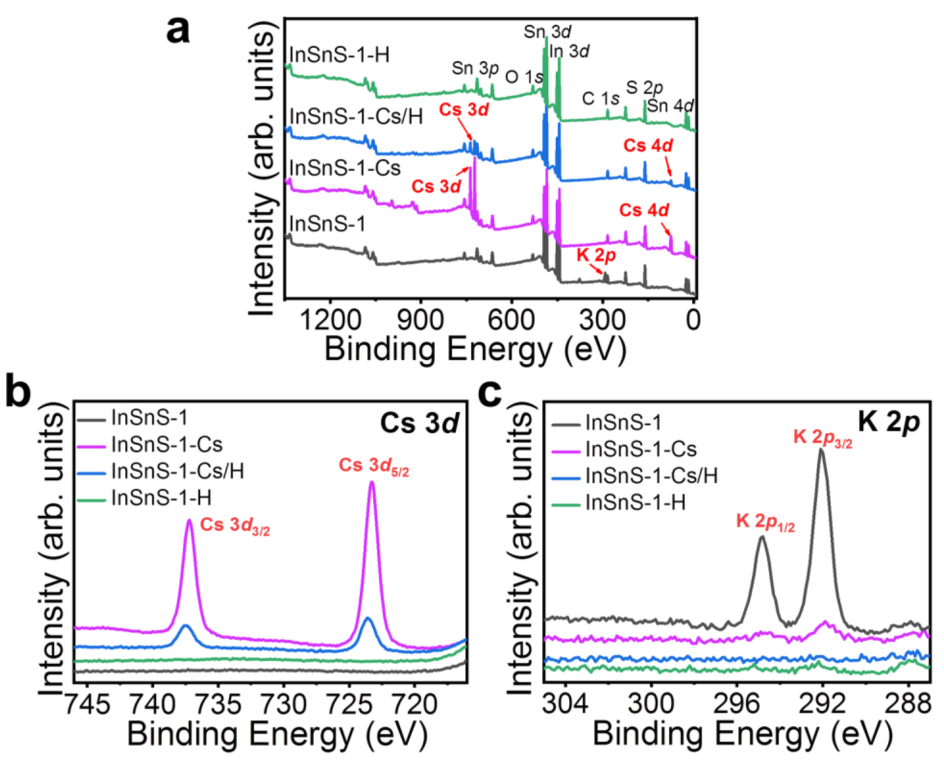

Supplementary Figure 10. (a) X-ray photoelectron spectra of InSnS-1, InSnS-1-Cs, InSnS-1-Cs/H and InSnS-1-H; narrow scan XPS spectra of Cs 3d (b) and K 2p (c) of InSnS-1, InSnS-1-Cs, InSnS-1-Cs/H and InSnS-1-H.

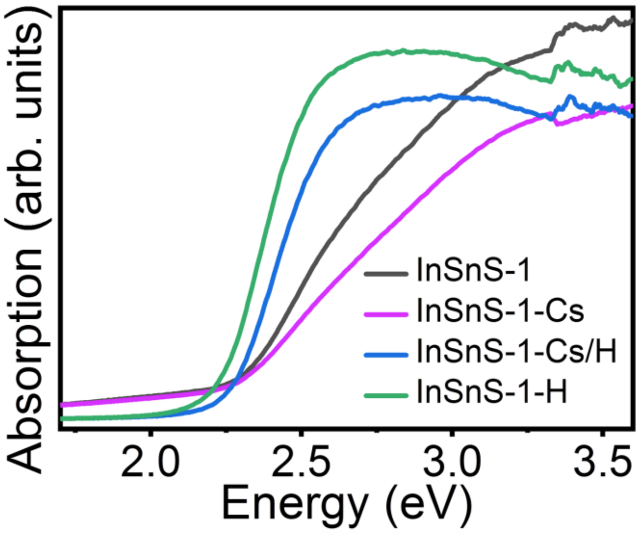

Supplementary Figure 11. Optical absorption spectra of InSnS-1, InSnS-1-Cs, InSnS-1-Cs/H and InSnS-1-H.

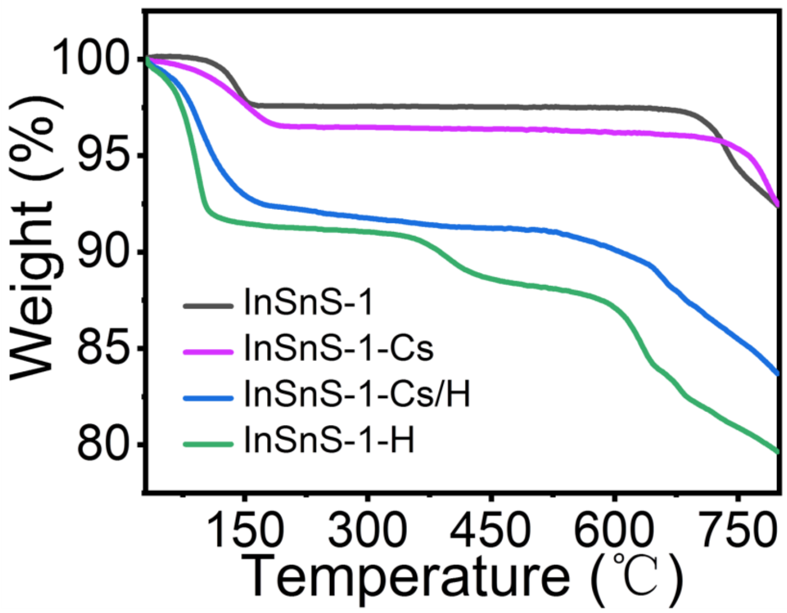

Supplementary Figure 12. TG curves of InSnS-1, InSnS-1-Cs, InSnS-1-Cs/H and InSnS-1-H.

The weight loss before 200 ℃ originates from a small amount of water adsorbed on the surface. A similar phenomenon has been also found in KMS-1. The increased weight loss of InSnS-1-Cs might be due to the increased water uptake after immersion in the solution. With the entering of H_3O^+ ions, the weight loss further increased and InSnS-1-H became less stable, probably because H^+ ions were combined with S to produce H_2S.

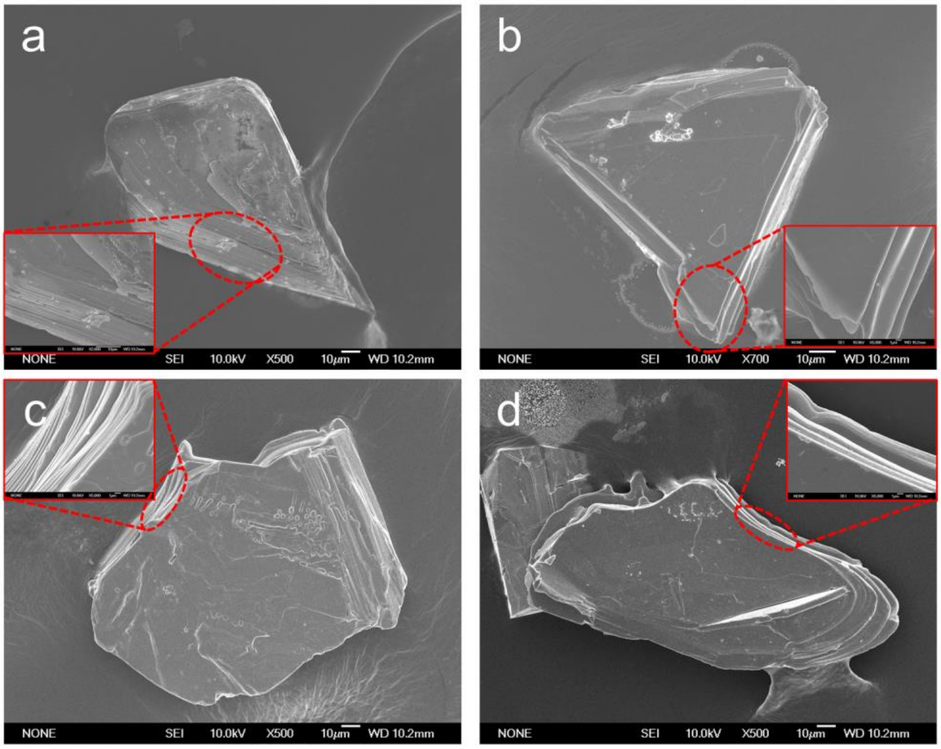

Supplementary Figure 13. SEM images of (a) InSnS-1, (b) InSnS-1-Cs, (c) InSnS-1-Cs/H and (d) InSnS-1-H crystals. Insets are partial enlargement images of the corresponding samples.

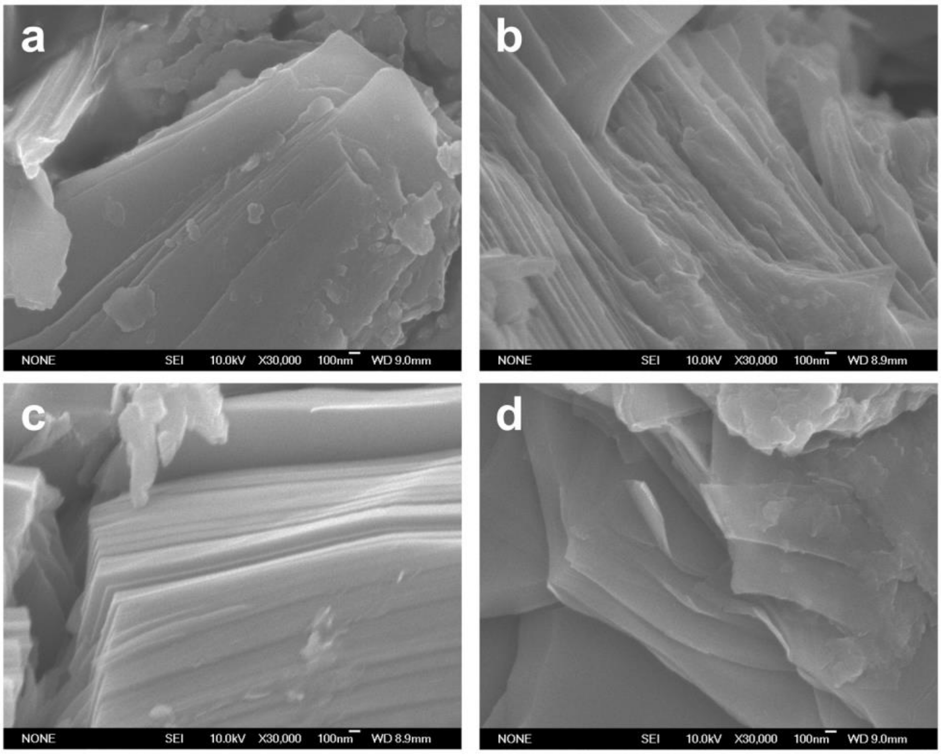

Supplementary Figure 14. SEM images of ground (a) InSnS-1, (b) InSnS-1-Cs, (c) InSnS-1-Cs/H and (d) InSnS-1-H polycrystalline samples.

Supplementary Table 8. Leaching percentages of Sn and In for InSnS-1 immersed in different concentrations of nitric acid solutions (*V*/*m* = 1000 mL/g at room temperature and 10 h contact time).

C_{HNO_3}(mol/L)	Leaching percentage (%)	
	In	Sn
0.1	0	0.06
0.3	0.03	0.05
0.5	0.13	0.03
1	0.79	0.00
2	1.44	0.00
3	1.62	0.00
4	2.79	0.03
5	23.74	66.19

Supplementary Table 9. Experimental results on the kinetics of Cs^+ ions capture by InSnS-1 in neutral and 1 mol/L HNO_3 solutions (V/m = 1000 mL/g at RT).

t (min)	Neutral		1mol/L HNO_3	
	C_e (mg/L)	R%	C_e (mg/L)	R%
0	3.44	0.00	4.3	0.00
2	0.42	87.79	1.24	71.16
5	0.3	91.28	1.12	73.95
10	0.22	93.60	0.96	77.67
15	0.2	94.19	0.88	79.53
20	0.2	94.19	0.76	82.33
30	0.196	94.30	0.68	84.19
60	0.162	95.29	0.7	83.72
120	0.14	95.93	0.74	82.79
180	0.138	95.99	0.76	82.33
360	0.136	96.05	0.8	81.40
720	0.144	95.81	0.92	78.60

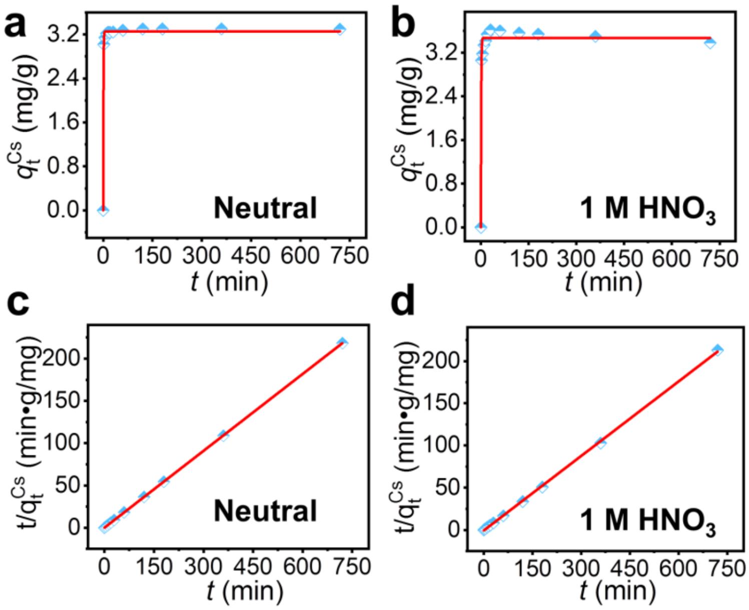

Supplementary Figure 15. Pseudo-first-order kinetic model fitting curves for kinetic data of Cs^+ ions capture by InSnS-1 in (a) neutral and (b) 1 mol/L HNO_3 solutions (red line: pseudo-first-order kinetic model). Pseudo-second-order kinetic model fitting curves for kinetic data of Cs^+ ions capture by InSnS-1 in (c) neutral and (d) 1 mol/L HNO_3 solutions (red line: pseudo-second-order kinetic model).

Supplementary Table 10. Kinetic fitting parameters for the capture of Cs^+ ions by InSnS-1 in neutral and 1 mol/L HNO_3 solutions (V/m = 1000 mL/g at room temperature and 4 h contact time).

	Pseudo-first-order model			Pseudo-second-order model		
	k_1 (min^{-1})	q_e (mg L^{-1})	R^2	k_2 (g mg^{-1} min^{-1})	q_e (mg L^{-1})	R^2
Neutral	1.2976	3.2574	0.9977	1.77846	3.2988	1
1 mol/L HNO_3	1.0285	3.4729	0.9857	-0.14479	3.3987	0.9997

Supplementary Table 11. Isothermal adsorption experimental results of Cs^+ capture by InSnS-1 in neutral and 1 mol/L HNO_3 solutions (V/m = 1000 mL/g at room temperature and 4 h contact time).

C_0 (mg/L)	C_e (mg/L)	q_e (mg/g)	C_0 (mg/L)	C_e (mg/L)	q_e (mg/g)	C_0 (mg/L)	C_e (mg/L)	q_e (mg/g)
Neutral			Neutral			1 mol/L HNO_3		
44.7	2.4	42.2	338.0	128.0	210.0	15.5	4.15	11.35
64.0	6.5	57.5	540.0	315.0	225.0	39.0	12	27
72.0	7.1	64.9	582.5	353.3	229.3	64.0	27	37
109.0	18.0	91.0	777.5	532.5	245.0	136.0	80	56
112.0	18.9	93.2	1050.0	795.0	255.0	176.0	110	66
167.0	43.3	123.8	1615.0	1345.0	270.0	264.	180	84
220.0	66.0	154.0	1904.0	1623.0	281.0	435	350	85
384.0	185.0	199.0				675	590	85

Supplementary Table 12. Isotherm fitting parameters for Cs^+ capture by InSnS-1 in neutral and 1 mol/L HNO_3 solutions (V/m = 1000 mL/g at room temperature and 4 h contact time).

	Langmuir model			Langmuir-Freundlich model			
	q_m (mg g^{-1})	b (L mg^{-1})	R^2	q_m (mg g^{-1})	b (L mg^{-1})	n	R^2
Neutral	279.75	0.01645	0.9687	316.04	0.01419	1.6096	0.9918
1 mol/L HNO_3	92.92	0.02575	0.9746	98.57	0.02217	1.1670	0.9780

Supplementary Table 13. Distribution coefficients and removal rates of Cs^+ ions captured by InSnS-1 in solutions with different pH values or different nitric acid concentrations (V/m = 1000 mL/g and 4 h contact time, RT).

pH or C_{HNO_3}	C_0 (mg/L)	C_e (mg/L)	K_d (mL/g)	R %
3 mol/L HNO_3	4.78	3.01	5.88×10^2	37.03
2 mol/L HNO_3	4.80	1.82	1.64×10^3	62.08
0.02	4.76	1.11	3.28×10^3	76.62
1.14	4.74	0.280	1.59×10^4	94.09
2.24	4.80	0.247	1.84×10^4	94.85
3.21	4.66	0.185	2.42×10^4	96.03
4.01	4.72	0.093	4.98×10^4	98.03
5.4	4.64	0.107	4.24×10^4	97.69
6.2	4.62	0.077	5.90×10^4	98.33
7.22	4.68	0.103	4.44×10^4	97.80
7.68	4.60	0.066	6.87×10^4	98.57
9.0	4.70	0.105	4.38×10^4	97.77
10.11	4.64	0.072	6.34×10^4	98.45
11.14	4.72	0.190	2.38×10^4	95.97
12.04	4.88	2.64	8.48×10^2	45.90

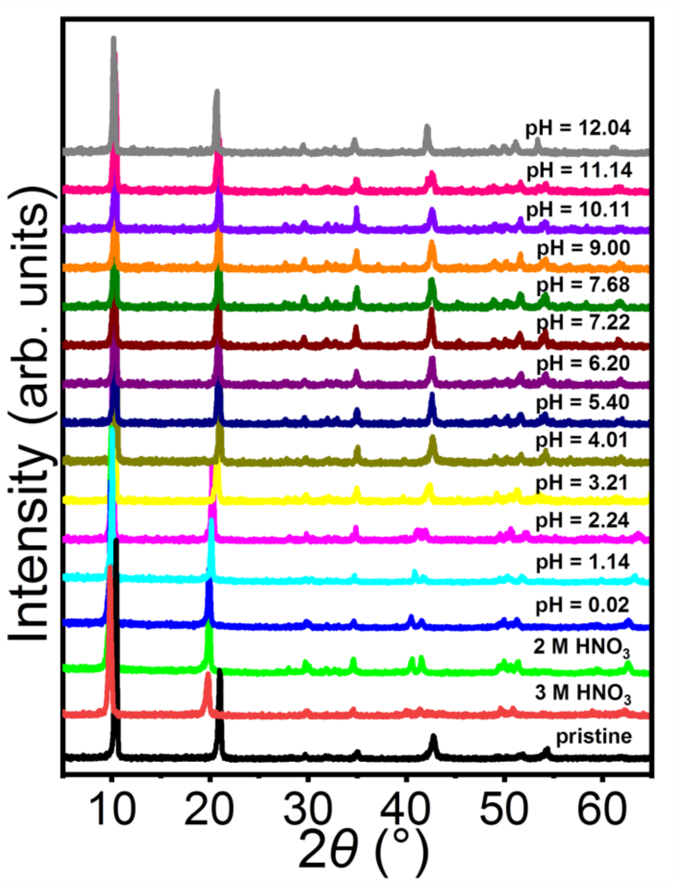

Supplementary Figure 16. PXRD patterns of InSnS-1 at various initial pH values (C_0 in the range of 4.6-4.88 mg/L for Cs^+ ions, V/m = 1000 mL/g, at room temperature).

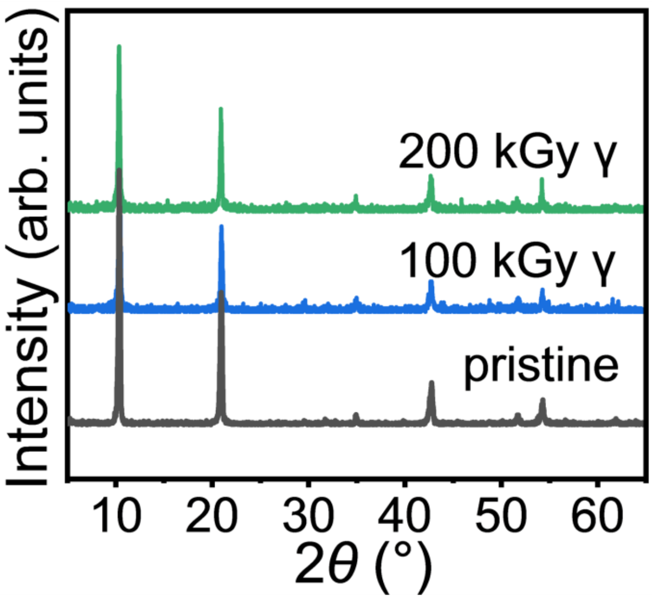

Supplementary Figure 17. PXRD pattern of InSnS-1 samples before and after irradiation.

Supplementary Table 14. Distribution coefficients and removal rates of Cs^+ ions captured by InSnS-1 samples before and after irradiation (V/m = 1000 mL/g at room temperature and 4 h contact time).

	C_0 (mg/L)	C_e (mg/L)	R (%)	K_d (mL/g)
Pristine	40.68	3.60	91.15	1.03×10^4
200 kGy	40.68	3.40	91.64	1.10×10^4
100 kGy	40.68	3.45	91.52	1.08×10^4

Supplementary Table 15. Results of competitive adsorption of Cs^+ and Na^+ ions by InSnS-1 in neutral, 1 mol/L HNO_3 and 3 mol/L HNO_3 solutions with different Na/Cs molar ratios (V/m = 1000 mL/g and 4 h contact time, at RT).

C_0^{Cs} (mg/L)	C_0^{Na} (mg/L)	Molar ratio (Na/Cs)	C_e^{Cs} (mg/L)	C_e^{Na} (mg/L)	R^{Cs} (%)	K_d^{Cs} (mL/g)	K_d^{Na} (mL/g)	$SF_{Cs/Na}$
Neutral								
4.12	33.9	47.5	0.358	25.5	91.32	1.05×10^4	3.27×10^2	32.17
4.40	82.3	108	1.04	64.4	76.35	3.23×10^3	2.78×10^2	11.62
4.46	147	191	1.63	126	63.52	1.74×10^3	1.65×10^2	10.58
4.43	291	380	2.20	265	50.37	1.01×10^3	98.6	10.30
4.47	458	591	2.65	430	40.69	6.86×10^2	65.5	10.47
4.54	742	945	3.11	720	31.61	4.62×10^2	31.7	14.58
4.54	1.47×10^3	1.87×10^3	3.65	1.44×10^3	19.51	2.42×10^2	19.5	12.44
4.56	2.32×10^3	2.94×10^3	3.98	2.29×10^3	12.88	1.48×10^2	15.2	9.72
4.62	4.59×10^3	5.75×10^3	4.31	4.59×10^3	6.77	72.6	1.64	44.26
3.508	1.12×10^4	1.84×10^4	3.38	1.10×10^4	3.64	37.7	17.8	2.12
1 mol/L HNO_3								
4.41	145	191	0.895	142	79.71	3.93×10^3	22.8	172.34
4.42	296	387	0.878	285	80.16	4.04×10^3	36.9	109.57
4.43	753	983	0.928	736	79.05	3.78×10^3	22.7	166.22
4.46	1.48×10^3	1.92×10^3	1.41	1.44×10^3	68.35	2.16×10^3	24.3	89.04
4.45	2.36×10^3	3.07×10^3	3.38	2.30×10^3	24.07	3.17×10^2	25.8	12.30
4.56	4.69×10^3	5.93×10^3	4.39	4.57×10^3	3.89	40.5	24.7	1.64
4.57	1.13×10^4	1.43×10^4	4.50	1.11×10^4	1.59	16.1	21.0	0.77
3 mol/L HNO_3								
4.48	272	351	2.78	257	37.98	6.12×10^2	59.8	10.24
4.49	709	912	2.78	674	38.23	6.19×10^2	51.9	11.94
4.46	2.25×10^3	2.92×10^3	2.81	2.18×10^3	37.11	5.90×10^2	30.2	19.52
4.55	4.59×10^3	5.84×10^3	3.14	4.37×10^3	31.06	4.51×10^2	51.5	8.75
4.43	1.14×10^4	1.49×10^4	4.38	1.07×10^4	1.07	10.9	69.2	0.16

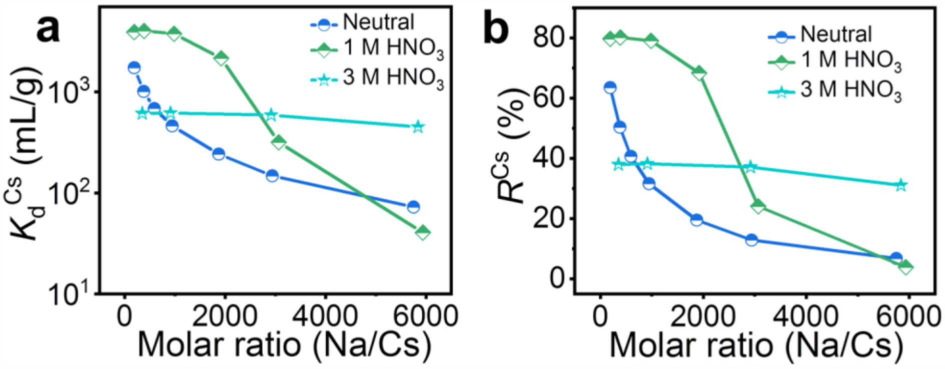

Supplementary Figure 18. Comparison of (a) K_d^{Cs} and (b) R^{Cs} values for the capture of Cs^+ ions by InSnS-1 in neutral, 1 mol/L HNO_3, and 3 mol/L HNO_3 solutions at different Na/Cs molar ratios from 190 to 6000 (*V*/*m* = 1000 mL/g and 4 h contact time, at RT).

Supplementary Table 16. Results of competitive adsorption of Cs^+ and Sr^{2+} ions by InSnS-1 in neutral, 1 mol/L HNO_3 and 3 mol/L HNO_3 solutions with different Sr/Cs molar ratios (*V*/*m* = 1000 mL/g at room temperature and 4 h contact time).

C_0^{Cs} (mg/L)	C_0^{Sr} (mg/L)	Molar ratio (Sr/Cs)	C_e^{Cs} (mg/L)	C_e^{Sr} (mg/L)	R^{Cs} (%)	K_d^{Cs} (mL/g)	R^{Sr} (%)	K_d^{Sr} (mL/g)	$SF_{Cs/Sr}$
Neutral									
4.77	4.52	1.44	0.106	0.665	97.78	4.40×10^4	85.29	5.80×10^3	7.59
4.74	46.5	14.9	0.886	0.536	81.31	4.35×10^3	98.85	8.57×10^4	0.05
4.63	487	160	4.27	388	7.78	84.3	20.44	2.57×10^2	0.33
1 mol/L HNO_3									
4.86	4.72	1.47	0.840	4.60	82.72	4.79×10^3	2.54	26.1	183.45
4.85	47.1	14.7	0.847	46.7	82.54	4.73×10^3	0.74	7.49	630.87
4.65	488	159	0.837	469	82.00	4.56×10^3	3.95	41.2	110.70
3 mol/L HNO_3									
4.89	4.77	1.48	2.85	4.75	41.72	7.16×10^2	0.42	4.21	170.00
4.82	46.9	14.8	2.85	46.7	40.87	6.91×10^2	0.36	3.64	189.97
4.65	491	160	2.43	461	47.74	9.14×10^2	6.15	65.6	13.93

Supplementary Table 17. Results of competitive adsorption of Cs^+ and La^{3+} ions by InSnS-1 in neutral, 1 mol/L HNO_3 and 3 mol/L HNO_3 solutions with different La/Cs molar ratios (V/m = 1000 mL/g at room temperature and 4 h contact time).

C_0^{Cs} (mg/L)	C_0^{La} (mg/L)	Molar ratio (La/Cs)	C_e^{Cs} (mg/L)	C_e^{La} (mg/L)	R^{Cs} (%)	K_d^{Cs} (mL/g)	R^{La} (%)	K_d^{La} (mL/g)	$SF_{Cs/La}$
Neutral									
1.93	4.23	2.09	0.0400	0.0100	97.93	4.74×10^4	99.76	4.14×10^5	0.11
2.00	45.5	21.8	0.375	0.0930	81.23	4.33×10^3	99.80	4.89×10^5	0.01
1.96	446	217	1.96	368	0.20	2.04	17.49	2.12×10^2	0.01
1 mol/L HNO_3									
1.92	4.63	2.30	0.413	4.54	78.51	3.65×10^3	1.94	19.8	184.31
1.95	43.8	21.5	0.409	42.6	79.05	3.77×10^3	2.74	28.2	133.93
1.93	448	222	0.736	425	61.81	1.62×10^3	5.13	54.1	29.90
3 mol/L HNO_3									
1.94	4.97	2.45	1.30	4.88	32.78	4.88×10^2	1.81	18.4	26.45
1.97	44.1	21.4	1.27	43.6	35.41	5.48×10^2	1.13	11.5	47.81
1.99	454	219	1.26	445	36.78	5.82×10^2	1.98	20.2	28.76

Supplementary Table 18. Results of competitive adsorption of Cs^{+}, Sr^{2+} and La^{3+} ions by InSnS-1 in neutral, 1 mol/L HNO_3 and 3 mol/L HNO_3 solutions with different Sr/La/Cs molar ratios (V/m = 1000 mL/g at room temperature and 4 h contact time).

C_0^{Cs} (mg/L)	C_0^{La} (mg/L)	C_0^{Sr} (mg/L)	Molar ratio (Sr:La:Cs)	C_e^{Cs} (mg/L)	C_e^{La} (mg/L)	C_e^{Sr} (mg/L)	R^{Cs} %	K_d^{Cs} (mL/g)	R^{La} %	K_d^{La} (mL/g)	R^{Sr} %	K_d^{Sr} (mL/g)	$SF_{Cs/La}$	$SF_{Cs/Sr}$
Neutral														
5.21	6.89	4.75	1.38:1.27:1	0.253	0.128	0.007	95.14	1.96×10^4	98.15	5.30×10^4	99.85	6.50×10^5	0.37	0.03
5.12	45.9	46.8	13.9:8.58:1	4.48	0.695	10.8	12.40	1.42×10^2	98.49	6.50×10^4	77.01	3.35×10^3	0.00	0.04
5.72	441	463	123:73.9:1	5.58	360	434	2.43	24.9	18.44	2.26×10^2	6.26	66.8	0.11	0.37
1 mol/L HNO_3														
4.94	4.77	4.77	1.47:0.925:1	1.35	4.55	4.72	72.58	2.65×10^3	4.72	49.5	1.05	10.6	53.47	249.89
5.13	45.4	46.0	13.6:8.45:1	1.40	43.2	45.8	72.71	2.66×10^3	4.83	50.7	0.43	4.37	52.50	610.01
5.66	433	454	122:73.1:1	3.00	391	449	47.11	8.91×10^2	9.57	1.06×10^2	1.10	11.1	8.42	80.00
3 mol/L HNO_3														
5.05	4.64	4.83	1.45:0.880:1	4.30	4.62	4.80	14.87	1.75×10^2	0.43	4.33	0.62	6.25	40.38	27.94
5.22	45.6	46.5	13.5:8.36:1	4.39	43.7	46.2	15.94	1.90×10^2	4.12	43.0	0.65	6.49	4.41	29.20
5.50	424	445	123:73.7:1	4.45	422	422	19.10	2.36×10^2	0.47	4.74	5.17	54.5	49.80	4.33

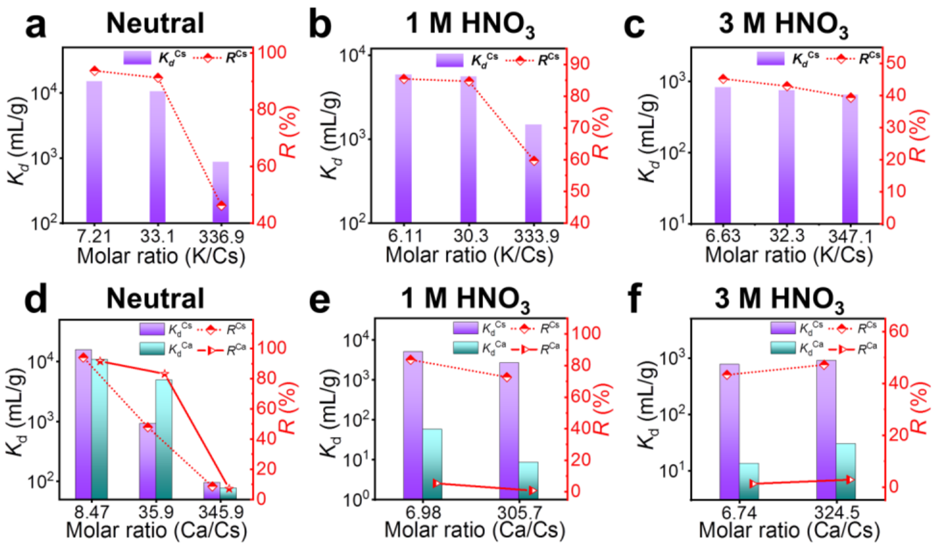

Supplementary Figure 19. K_d and R of Cs^+ ion removed by InSnS-1 in (a) neutral, (b) 1 mol/L HNO_3 and (c) 3 mol/L HNO_3 solutions with different K/Cs molar ratios. K_d and R of Cs^+ and Ca^{2+} ions removed by InSnS-1 in (d) neutral, (e) 1 mol/L HNO_3 and (f) 3 mol/L HNO_3 solutions with different Ca/Cs molar ratios.

InSnS-1 showed excellent selectivity for Cs^+ in neutral solutions under the K/Cs molar ratio of 7.21 and 33.1 (K_d^{Cs} of 1.50×10^4 mL/g and 1.05×10^4 mL/g, respectively, R^{Cs} of 93.75% and 91.29%, respectively). When the concentration of K^+ increased (K/Cs molar ratio of 336.9), the capture performance of InSnS-1 for Cs^+ decreased significantly (K_d^{Cs} of 8.60×10^2 mL/g and R^{Cs} of 46.23%). While in 1 mol/L HNO_3 solution, even when the K/Cs molar ratio reached 333.9, K_d^{Cs} and R^{Cs} could still reach 1.48×10^3 mL/g and 59.65%, respectively. It should be noted that in acidic solutions, especially 3 mol/L HNO_3 solutions, the effect of elevated K^+ ion concentration on Cs^+ capture performance is significantly smaller than that in neutral solutions. Under neutral conditions, when the concentrations of Cs^+ and competing Ca^{2+} were low (*i.e.*, the Ca/Cs molar ratio was 8.47), InSnS-1 exhibited high K_d and R for Cs^+ and Ca^{2+} ions (K_d^{Cs} of 1.57×10^4 mL/g, R^{Cs} of 94.01%, K_d^{Ca} of 1.08×10^4 mL/g and R^{Ca} of 91.54%). In 1 mol/L HNO_3 solution, K_d^{Cs} still reached 2.67×10^3 mL/g with R^{Cs} of 72.73% and $SF_{Cs/Ca}$ of 316.09 even under very high concentrations of Ca^{2+} (*i.e.*, the Ca/Cs molar ratio was 305.7). Conversely, the removal performance of InSnS-1 for Ca^{2+} in 1 mol/L HNO_3 was significantly lower under the Ca/Cs molar ratios of 6.98 and 305.7, with K_d^{Ca} of 57.0 mL/g and 8.44 mL/g, respectively, and R^M of 5.39% and 0.84%, respectively. Even in 3 mol/L HNO_3 solutions containing high concentrations of Ca^{2+}, InSnS-1 still reached R^{Cs} of 47.21%, while R^{Ca} was only 2.87%. The above results are similar to that for competing ion experiments (Na/Cs, Sr/Cs, La/Cs, La/Sr/Cs), all reflecting that the acidic conditions are favorable for the selective capture of Cs^+ by InSnS-1.

Supplementary Table 19. Results of competitive adsorption of Cs^+ ion by InSnS-1 in neutral, 1 mol/L HNO_3 and 3 mol/L HNO_3 solutions with different K/Cs molar ratios (V/m = 1000 mL/g at room temperature and 4 h contact time).

C_0^{Cs} (mg/L)	C_0^{K} (mg/L)	Molar ratio (K/Cs)	C_e^{Cs} (mg/L)	R^{Cs} (%)	K_d^{Cs} (mL/g)
Neutral					
4.347	9.214	7.21	0.272	93.75	1.50×10^4
4.391	42.813	33.1	0.382	91.29	1.05×10^4
4.100	442.515	366.9	2.205	46.23	8.60×10^2
1 mol/L HNO_3					
5.095	9.154	6.11	0.742	85.44	5.87×10^3
4.863	43.318	30.3	0.744	84.69	5.53×10^3
4.589	450.788	333.9	1.852	59.65	1.48×10^3
3 mol/L HNO_3					
4.680	9.124	6.63	2.562	45.25	8.27×10^2
4.611	43.886	32.3	2.630	42.96	7.53×10^2
4.408	450.135	347.1	2.671	39.41	6.50×10^2

Supplementary Table 20. Results of competitive adsorption of Cs^+ and Ca^{2+} ions by InSnS-1 in neutral, 1 mol/L HNO_3 and 3 mol/L HNO_3 solutions with different Ca/Cs molar ratios (V/m = 1000 mL/g at room temperature and 4 h contact time).

C_0^{Cs} (mg/L)	C_0^{Ca} (mg/L)	Molar ratio (Ca/Cs)	C_e^{Cs} (mg/L)	C_e^{Ca} (mg/L)	R^{Cs} (%)	K_d^{Cs} (mL/g)	R^{Ca} (%)	K_d^{Ca} (mL/g)	$SF_{Cs/Ca}$
Neutral									
4.148	10.596	8.47	0.248	0.896	94.01	1.57×10^4	91.54	1.08×10^4	1.45
4.024	43.541	35.9	2.099	7.319	47.83	9.17×10^2	83.19	4.95×10^3	0.19
3.934	410.324	345.9	3.596	381.272	8.61	94.2	7.08	76.2	1.24
1 mol/L HNO_3									
4.470	9.409	6.98	0.727	8.901	83.73	5.15×10^3	5.39	57.0	90.30
4.432	408.583	305.7	1.209	405.165	72.73	2.67×10^3	0.84	8.44	316.09
3 mol/L HNO_3									
4.474	9.087	6.74	2.535	8.969	43.34	7.65×10^2	1.30	13.1	58.17
4.391	429.697	324.5	2.318	417.371	47.21	8.94×10^2	2.87	29.5	30.28

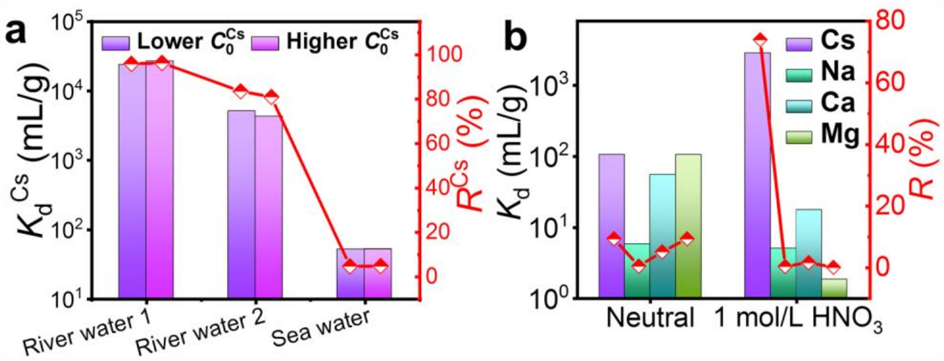

Supplementary Figure 20. Distribution coefficients and removal rates of Cs^+ ions captured by InSnS-1 in (a) different actual water samples (River water 1 was taken from Longyan, Fujian; river water 2 was taken from Fuzhou, Fujian; sea water was taken from Gulangyu, Xiamen, Fujian). (b) Distribution coefficients and removal rates of Cs^+, Na^+, Ca^{2+}, Mg^{2+} ions captured by InSnS-1 in neutral and 1 mol/L HNO_3 solutions with high concentration of Na^+, Ca^{2+}, Mg^{2+} ions and low concentration of Cs^+ ion. (Coloured bars: K_d; red dotted line: R.)

Supplementary Table 21. Distribution coefficients and removal rates of Cs^+ ions captured by InSnS-1 in different actual water samples (V/m = 1000 mL/g at room temperature and 4 h contact time).

Water samples	C_0^M (mg/L)					C_e^{Cs}	R^{Cs} (%)	K_d^{Cs}
	K^+	Na^+	Ca^{2+}	Mg^{2+}	Cs^+	(mg/L)		(mL/g)
River water 1	2.85	5.03	7.31	1.12	5.99	0.240	95.99	2.39×10^4
(Longyan, Fujian)	2.77	4.52	6.71	0.980	15.5	0.558	96.40	2.68×10^4
River water 2	3.69	16.7	11.0	1.92	4.04	0.663	83.60	5.10×10^3
(Fuzhou, Fujian)	3.62	16.5	11.0	1.90	8.94	1.69	81.06	4.28×10^3
Sea water	310	8.71×10^3	377	992	0.975	0.928	4.87	51.2
(Gulangyu, Xiamen, Fujian)	311	8.65×10^3	370	989	8.33	7.93	4.91	51.6

Supplementary Table 22. Distribution coefficients and removal rates of Cs^+, Na^+, Ca^{2+}, Mg^{2+} ions captured by **InSnS-1** in neutral, 1 mol/L HNO_3 solutions with high concentration of Na^+, Ca^{2+}, Mg^{2+} ions and low concentration of Cs^+ ion (V/m = 1000 mL/g at room temperature and 4 h contact time).

Ions	C_0 (mg/L)	C_e (mg/L)	R (%)	K_d (mL/g)
Neutral				
Cs^+	4.76	4.31	9.45	1.04×10^2
Na^+	47.5	47.2	0.57	5.72
Ca^{2+}	39.6	37.6	5.20	54.9
Mg^{2+}	211	191	9.44	1.04×10^2
1 mol/L HNO_3				
Cs^+	4.55	1.18	73.98	2.84×10^3
Na^+	48.6	48.4	0.49	4.96
Ca^{2+}	41.1	40.4	1.70	17.3
Mg^{2+}	220	220	0.18	1.82

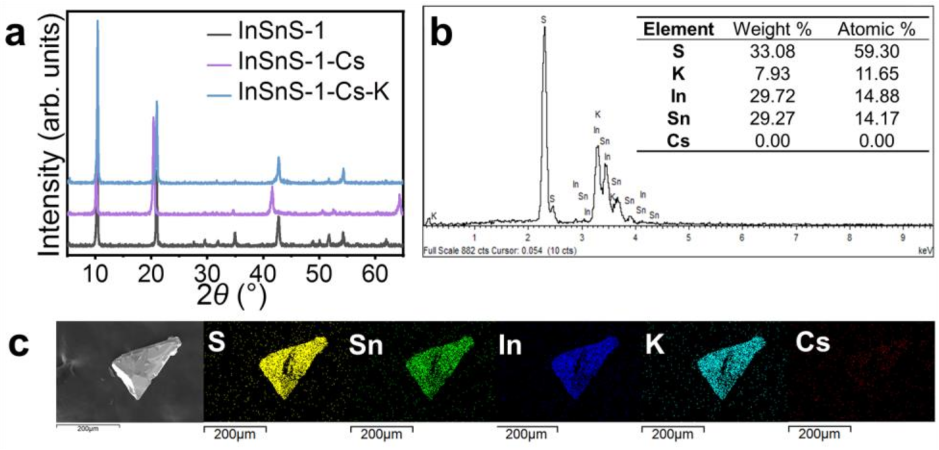

Supplementary Figure 21. (a) PXRD patterns of InSnS-1, InSnS-1-Cs and InSnS-1-Cs-K. (b) EDS analysis results of InSnS-1-Cs-K. (c) Elemental distribution map of InSnS-1-Cs-K.

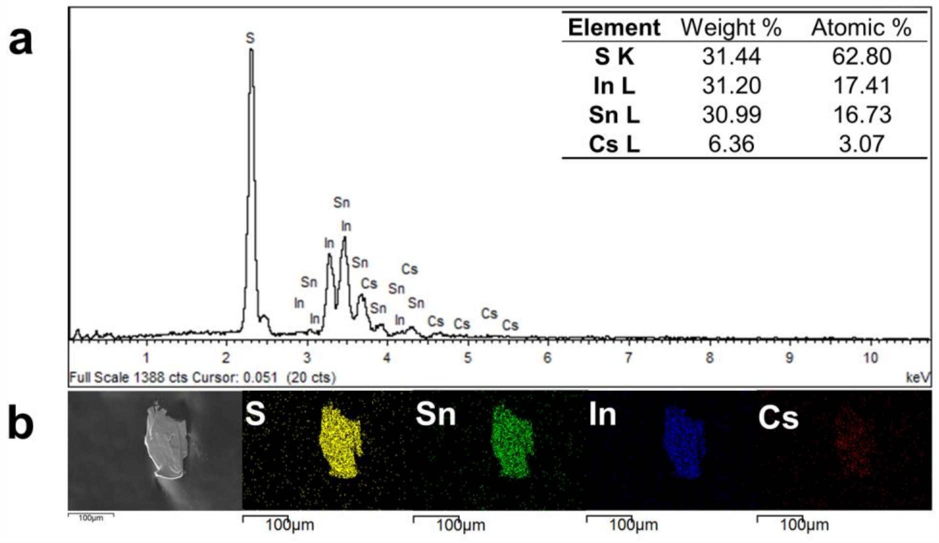

Supplementary Figure 22. (a) EDS analysis results of InSnS-1-Cs-H. (b) Elemental distribution map of InSnS-1-Cs-H.

Supplementary Table 23. Results of EDS analysis under different desorption conditions.

Experimental conditions	Results of EDS analysis (Atomic %)			
	In	Sn	S	Cs
10 mg **InSnS-1-Cs** + 10 mL 1 mol/L HNO_3, 6 h	16.91	17.19	61.55	4.36
10 mg **InSnS-1-Cs** + 10 mL 1 mol/L HNO_3, 12 h	17.41	16.73	62.80	3.07
10 mg **InSnS-1-Cs** + 10 mL 1 mol/L HNO_3, 6 h (renew the HNO_3 solution once)	17.46	19.37	60.76	2.42
10 mg **InSnS-1-Cs** + 10 mL 1 mol/L HNO_3, 6 h (renew the HNO_3 solution twice)	18.02	18.15	63.82	0.00

(Note: Equal time intervals for each renewal of the nitric acid solution. For example, renewing the solution twice means that the solution will be renewed once at 2 h and 4 h respectively.)

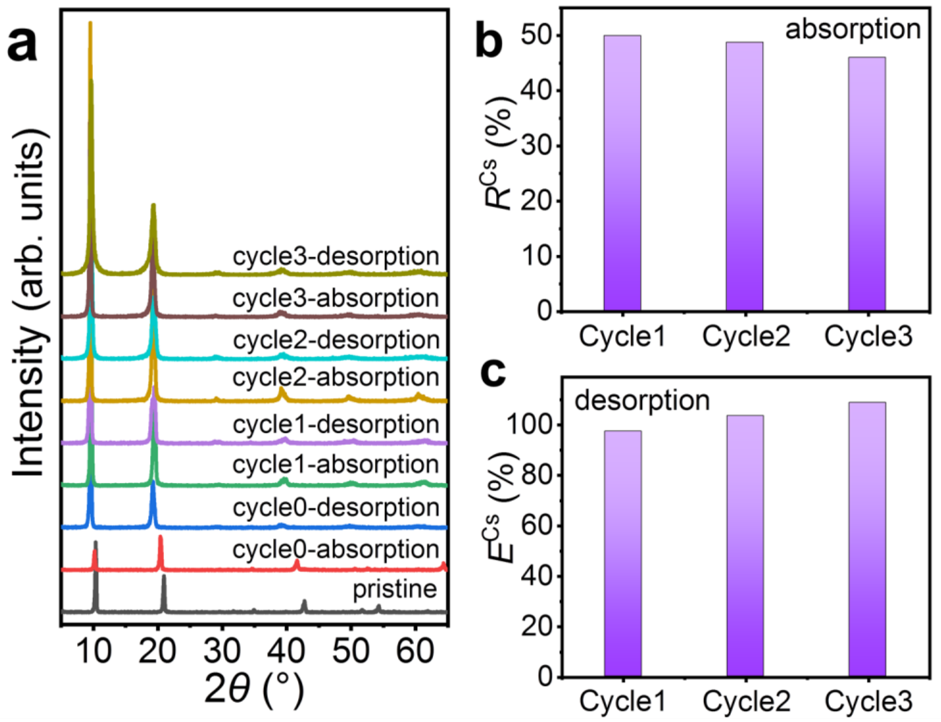

Supplementary Figure 23. (a) PXRD patterns of samples after each round of adsorption and desorption. (b) Adsorption rates (R^{Cs}) and (c) desorption rates (E^{Cs}) at each cycle (V/m = 1000 mL/g, at room temperature).

Supplementary Table 24. Results of cycling experiments.

Number of cycles	Adsorption					Desorption			
	m_1 (mg)	V_1 (mL)	C_0^{Cs} (mg/L)	C_e^{Cs} (mg/L)	R^{Cs} (%)	m_2 (mg)	V_2 (mL)	C_e^{Cs} (mg/L)	E^{Cs} (%)
1	172	172	88.4	44.25	49.94	150	150	41.45	97.44
2	123	123	88.4	45.30	48.76	99.5	99.5	43.05	103.57
3	77	77	88.4	47.75	45.98	57	57	42.75	108.83

(Note: m_1, V_1 are the mass of adsorbent and the corresponding volume of solution for the adsorption process, respectively. m_2, V_2 are the mass of samples and the corresponding volume of solution for the desorption process, respectively.)

Supplementary Table 25. Leaching of In, Sn, and S during the desorption in the cycle experiment.

Number of cycles	m_2 (mg)	V_2 (mL)	C_M (mg/L)			m_M (mg)		
			In	S	Sn	In	S	Sn
1	150	150	14.7	6.11	0.0130	2.21	0.916	0.002
2	99.5	99.5	17.3	3.51	0.0215	1.72	0.349	0.002
3	57	57	10.4	3.75	0.0315	0.591	0.214	0.002

(Note: C_M is the concentration of each ion in the solution after desorption; m_M is the leaching mass of each ion.)

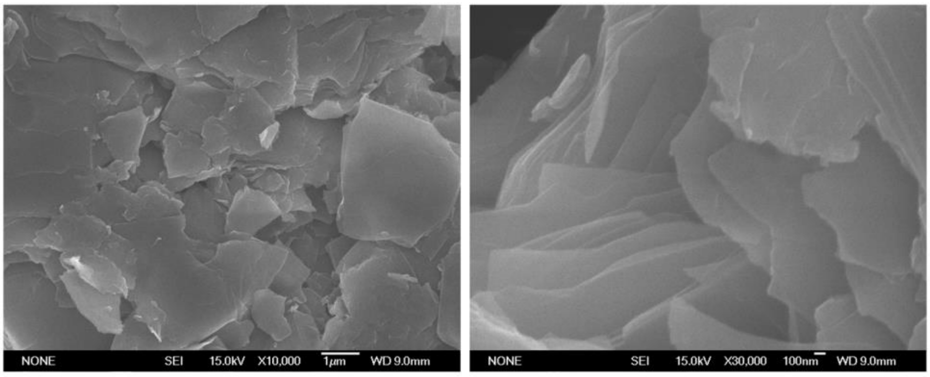

Supplementary Figure 24. SEM image of the sample after three cycles (The right image is a partial enlargement of the left image.).

Supplementary Table 26. Results of ion exchange column experiments under neutral conditions (C_0^{Cs} = 190.13 mg/L, room temperature).

t (min)	C_t (mg/L)	C_t/C_0	R^{Cs} (%)	t (min)	C_t (mg/L)	C_t/C_0	R^{Cs} (%)
2.5	0.05	0.0002	99.98	1197.5	1.10	0.0058	99.42
47.5	0.02	0.0001	99.99	1247.5	2.48	0.0130	98.70
97.5	0.02	0.0001	99.99	1297.5	5.40	0.0284	97.16
147.5	0.02	0.0001	99.99	1347.5	11.25	0.0592	94.08
197.5	0.02	0.0001	99.99	1397.5	21.00	0.1105	88.95
247.5	0.02	0.0001	99.99	1447.5	35.63	0.1874	81.26
297.5	0.03	0.0001	99.99	1497.5	53.78	0.2828	71.72
347.5	0.02	0.0001	99.99	1547.5	65.93	0.3467	65.33
397.5	0.02	0.0001	99.99	1597.5	45.15	0.2375	76.25
447.5	0.02	0.0001	99.99	1647.5	96.90	0.5097	49.03
497.5	0.02	0.0001	99.99	1697.5	113.18	0.5953	40.47
547.5	0.02	0.0001	99.99	1747.5	122.70	0.6454	35.46
597.5	0.02	0.0001	99.99	1797.5	132.15	0.6951	30.49
647.5	0.02	0.0001	99.99	1847.5	170.25	0.8955	10.45
697.5	0.02	0.0001	99.99	1897.5	174.00	0.9152	8.48
747.5	0.02	0.0001	99.99	1947.5	176.25	0.9270	7.30
797.5	0.02	0.0001	99.99	1997.5	174.75	0.9191	8.09
847.5	0.02	0.0001	99.99	2047.5	175.50	0.9231	7.69
897.5	0.02	0.0001	99.99	2097.5	179.25	0.9428	5.72
947.5	0.02	0.0001	99.99	2147.5	175.50	0.9231	7.69
997.5	0.32	0.0017	99.83	2197.5	174.75	0.9191	8.09
1047.5	0.35	0.0018	99.82	2247.5	174.75	0.9191	8.09
1097.5	0.41	0.0022	99.78	2297.5	176.25	0.9270	7.30
1147.5	0.57	0.0030	99.70				

Supplementary Table 27. Results of ion exchange column experiments under 1 mol/L HNO_3 conditions (C_0^{Cs} = 84.25 mg/L, room temperature).

t (min)	C_t (mg/L)	C_t/C_0	R^{Cs} (%)	*t* (min)	C_t (mg/L)	C_t/C_0	R^{Cs} (%)
2.5	0.12	0.0014	99.86	497.5	0.09	0.0011	99.89
22.5	0.09	0.0010	99.90	547.5	0.13	0.0015	99.85
42.5	0.15	0.0018	99.82	597.5	0.55	0.0065	99.35
62.5	0.55	0.0065	99.35	647.5	2.70	0.0320	96.80
82.5	0.10	0.0012	99.88	697.5	8.70	0.1033	89.67
102.5	0.11	0.0013	99.87	747.5	18.85	0.2237	77.63
122.5	0.13	0.0016	99.84	797.5	30.05	0.3567	64.33
142.5	0.08	0.0009	99.91	847.5	24.38	0.2893	71.07
167.5	0.09	0.0011	99.89	897.5	47.25	0.5608	43.92
197.5	0.10	0.0011	99.89	972.5	60.75	0.7211	27.89
247.5	0.08	0.0010	99.90	1047.5	75.25	0.8932	10.68
297.5	0.09	0.0010	99.90	1122.5	83.00	0.9852	1.48
347.5	0.08	0.0009	99.91	1197.5	85.50	1.0148	-1.48
397.5	0.14	0.0017	99.83	1272.5	90.75	1.0772	-7.72
447.5	0.11	0.0012	99.88	1347.5	87.50	1.0386	-3.86

Supplementary Table 28. Thomas model fitting parameters for ion exchange column experimental data.

Solutions	K_T (L min^{-1} mg^{-1})	q_e (mg/g)	R^2
190.13 mg/L Cs^+	4.00×10^{-5}	216.06	0.9941
1 mol/L HNO_3 with 84.25 mg/L Cs^+	1.39×10^{-4}	50.50	0.9950

Supplementary References

(1) Manos, M. J.; Ding, N. & Kanatzidis, M. G. Layered metal sulfides: Exceptionally selective agents for radioactive strontium removal. *Proc. Natl. Acad. Sci. U.S.A.* **105,** 3696-3699 (2008).

(2) Manos, M. J. & Kanatzidis, M. G. Highly efficient and rapid Cs^+ uptake by the layered metal sulfide $K_{2x}Mn_xSn_{3-x}S_6$ (KMS-1). *J. Am. Chem. Soc.* **131,** 6599-6607 (2009).

(3) Manos, M. J. & Kanatzidis, M. G. Layered metal sulfides capture uranium from seawater. *J. Am. Chem. Soc.* **134,** 16441-16446 (2012).

(4) Fard, Z. H.; Islam, S. M. & Kanatzidis, M. G. Porous Amorphous chalcogenides as selective adsorbents for heavy metals. *Chem. Mater.* **27,** 6189-6192 (2015).

(5) Fard, Z. H.; Malliakas, C. D.; Mertz, J. L. & Kanatzidis, M. G. Direct extraction of Ag^+ and Hg^{2+} from cyanide complexes and mode of binding by the layered $K_2MgSn_2S_6$ (KMS-2). *Chem. Mater.* **27,** 1925-1928 (2015).

(6) Xiao, C.; Hassanzadeh Fard, Z.; Sarma, D.; Song, T. B.; Xu, C. & Kanatzidis, M. G. Highly efficient separation of trivalent minor actinides by a layered metal sulfide ($KInSn_2S_6$) from acidic radioactive waste. *J. Am. Chem. Soc.* **139,** 16494-16497 (2017).

(7) Sarma, D.; Malliakas, C. D.; Subrahmanyam, K. S.; Islama, S. M. & Kanatzidis, M. G. $K_{2x}Sn_{4-x}S_{8-x}$ (x = 0.65~1): A new metal sulfide for rapid and selective removal of Cs^+, Sr^{2+} and UO_2^{2+} ions. *Chem. Sci.* **7,** 1121-1132 (2016).

(8) Manos, M. J.; Chrissafis, K. & Kanatzidis, M. G. Unique pore selectivity for Cs^+ and exceptionally high NH_4^+ exchange capacity of the chalcogenide material $K_6Sn[Zn_4Sn_4S_{17}]$. *J. Am. Chem. Soc.* **128,** 8875-8883 (2006).

(9) Wang, R. Q.; Chen, H. J.; Mao, Y.; Hadar, I.; Bu, K. J.; Zhang, X.; Pan, J.; Gu, Y. H.; Guo, Z. N.; Huang, F. Q. & Kanatzidis, M. G. $K_x[Bi_{4-x}Mn_xS_6]$, design of a highly selective ion exchange material and direct gap 2D semiconductor.

J. Am. Chem. Soc. **141,** 16903-16914 (2019).

(10) Zhang, R. C.; Yao, H. G.; Ji, S. H.; Liu, M. C.; Ji, M. & An, Y. L. Copper-rich framework sulfides: $A_4Cu_8Ge_3S_{12}$ (A = K, Rb) with cubic perovskite structure. *Inorg. Chem.* **49,** 6372-6374 (2010).

(11) Pogu, A.; Jaschin, P. W.; Varma, K. B. R. & Vidyasagar, K. Structural variants and characterization of $A_2CdSn_2S_6$ (A = Cs, Rb and K) compounds. *J. Solid State Chem.* **277,** 713-720 (2019).

(12) Yao, H. G.; Ji, M.; Ji, S. H.; Zhang, R. C.; An, Y. L. & Ning, G. L. Solvothermal syntheses of two novel layered quaternary silver-antimony(III) sulfides with different strategies. *Cryst. Growth Des.* **9,** 3821-3824 (2009).

(13) Wang, R.; Zhang, X.; He, J.; Zheng, C.; Lin, J. & Huang, F. Synthesis, crystal structure, electronic structure, and photoelectric response properties of KCu_2SbS_3. *Dalton Trans.* **45,** 3473-3479 (2016).

(14) Fabry, J.; Havlak, L.; Dusek, M.; Vanek, P.; Drahokoupil, J. & Jurek, K. Structure determination of $KLaS_2$, $KPrS_2$, $KEuS_2$, $KGdS_2$, $KLuS_2$, KYS_2, $RbYS_2$, $NaLaS_2$ and crystal-chemical analysis of the group 1 and thallium(I) rare-earth sulfide series. *Acta Crystallogr. Sect. B-Struct. Sci. Cryst. Eng. Mat.* **70,** 360-371 (2014).

(15) Baiyin, M.; An, Y. L.; Liu, X.; Ji, M.; Jia, C. Y. & Ning, G. L. $K_2Ag_6Sn_3S_{10}$: A quaternary sulfide composed of silver sulfide layers pillared by zigzag chains ${}_{\infty}^{1}[SnS_3]^{2-}$. *Inorg. Chem.* **43,** 3764-3765 (2004).

(16) Li, X.; Li, C.; Gong, P.; Lin, Z.; Yao, J. & Wu, Y. Syntheses, crystal structures and physical properties of three new chalcogenides: $NaGaGe_3Se_8$, $K_3Ga_3Ge_7S_{20}$, and $K_3Ga_3Ge_7Se_{20}$. *Dalton Trans.* **45,** 532-538 (2016).

(17) Ho, Y. S.; Wase, D. A. J. & Forster, C. F. Kinetic studies of competitive heavy metal adsorption by sphagnum Moss Peat. *Environ. Technol.* **17,** 71-77 (1996).

(18) Duong, D. D. *Adsorption Analysis: Equilibria and Kinetics* (1998).

(19) Mathialagan, T. & Viraraghavan, T. Adsorption of cadmium from aqueous solutions by perlite. *J. Hazard. Mater.* **94,** 291-303 (2002).

(20) Chen, Z.; Wu, Y.; Wei, Y. & Mimura, H. Preparation of silica-based titanate adsorbents and application for strontium removal from radioactive contaminated wastewater. *J. Radioanal. Nucl. Chem.* **307,** 931-940 (2016).
